# Comprehensive analysis of the diagnostic and therapeutic value of the hypoxia-related gene PLAUR in the progression of atherosclerosis

**DOI:** 10.1038/s41598-023-35548-z

**Published:** 2023-05-26

**Authors:** Chengyi Dai, Yuhang Lin

**Affiliations:** 1 The First People’s Hospital of Xiaoshan District, Xiaoshan First Affiliated Hospital of Wenzhou Medical University, Hangzhou, 311200 Zhejiang China; 2 Department of Neurology, Wenling First People’s Hospital, The Affiliated Wenling Hospital of Wenzhou Medical University, Wenling, 317500 Zhejiang China

**Keywords:** Computational biology and bioinformatics, Biomarkers, Cardiology

## Abstract

Atherosclerosis (AS) is a major contributor to a variety of negative clinical outcomes, including stroke and myocardial infarction. However, the role and therapeutic value of hypoxia-related genes in AS development has been less discussed. In this study, Plasminogen activator, urokinase receptor (PLAUR) was identified as an effective diagnostic marker for AS lesion progression by combining WGCNA and random forest algorithm. We validated the stability of the diagnostic value on multiple external datasets including humans and mice. We identified a significant correlation between PLAUR expression and lesion progression. We mined multiple single cell-RNA sequencing (sc-RNA seq) data to nominate macrophage as the key cell cluster for PLAUR mediated lesion progression. We combined cross-validation results from multiple databases to predict that HCG17-hsa-miR-424-5p-HIF1A, a competitive endogenous RNA (ceRNA) network, may regulate hypoxia inducible factor 1 subunit alpha (HIF1A) expression. The DrugMatrix database was used to predict alprazolam, valsartan, biotin A, lignocaine, and curcumin as potential drugs to delay lesion progression by antagonizing PLAUR, and AutoDock was used to verify the binding ability of drugs and PLAUR. Overall, this study provides the first systematic identification of the diagnostic and therapeutic value of PLAUR in AS and offers multiple treatment options with potential applications.

## Introduction

Atherosclerosis (AS), the most common cardiovascular disease (CVD), is characterized by the accumulation of lipids, cells, and extracellular matrix in the vascular intima leading to thickening and hardening of the arterial wall^1^. Lipid metabolism disturbance and local inflammation are the main contributors to AS, which has been generally recognized for more than half a century^2^. Current clinical interventions are designed to reduce blood lipids and control blood pressure through an improved lifestyle and regular medication, which have been shown to be effective in improving prevention and prognosis of AS^3^. However, they have proven unable to completely prevent the occurrence and development of AS^4^. For ideal preventive and therapeutic objectives, a deeper understanding of the molecular pathways underlying the emergence of AS is therefore necessary.

There is increasing evidence that there are regions of severe hypoxia within AS plaques, especially at sites with abundant macrophage infiltration^5^. Hypoxia theory contends that the development of AS is influenced by an imbalance between the supply and demand of oxygen in the arterial wall^6^. This hypoxia microenvironment has the potential to fundamentally alter the biological properties of many cell types involved in the development of AS plaques. This may determine whether the plaques evolve into a stable or unstable phenotype. This is hypothesized to be mediated by several different possible pathways, including alterations in lipid homeostasis, increased inflammation, and changes in angiogenesis^7^. Therefore, dissecting the hypoxia environment in AS plaques can help develop therapeutic interventions that may be beneficial for AS. Stabilization of HIF-1α is a hallmark of hypoxia, and direct evidence of HIF-1α expression was seen in plaque tissue derived from carotid and femoral endarterectomy, strongly suggesting that HIF-1α may be involved in the development of AS^8^. HIF-1α is involved in the transcriptional activation of numerous target genes during hypoxia, some of which have been demonstrated to be intimately associated with the development of AS^9^. In recent years, the boom of transcriptomic research has helped researchers deepen understanding of the development mechanism of AS, and has shown good prospects for application in the exploration of biomarkers and therapeutic targets^10,11^. Therefore, we hope to find candidate genes for the prediction of AS progression and as possible therapeutic targets through data mining.

In this study, existing public resources were mined based on the comprehensive bioinformatics method. Combining the results of differentially expressed genes (DEG) analysis, weighted gene co-expression network analysis (WGCNA) and random forest analysis identified PLAUR as a key hypoxia-related gene involved in plaque progression. Chronic inflammation and immune cell infiltration are prominent pathological features of AS. Therefore, we further explored the hypoxia microenvironment and immune infiltration of these samples and the correlation of different types of immune cells with PLAUR. Furthermore, we predicted the ceRNA network that regulates PLAUR, which provides a basis for subsequent studies. Finally, 5 drugs targeting PLAUR were chosen from the DrugMatrix database and validated using Autodock software, which may help enrich the drug treatments for AS.

## Materials and methods

### Microarray data and data pre-processing

**Figure 1:**
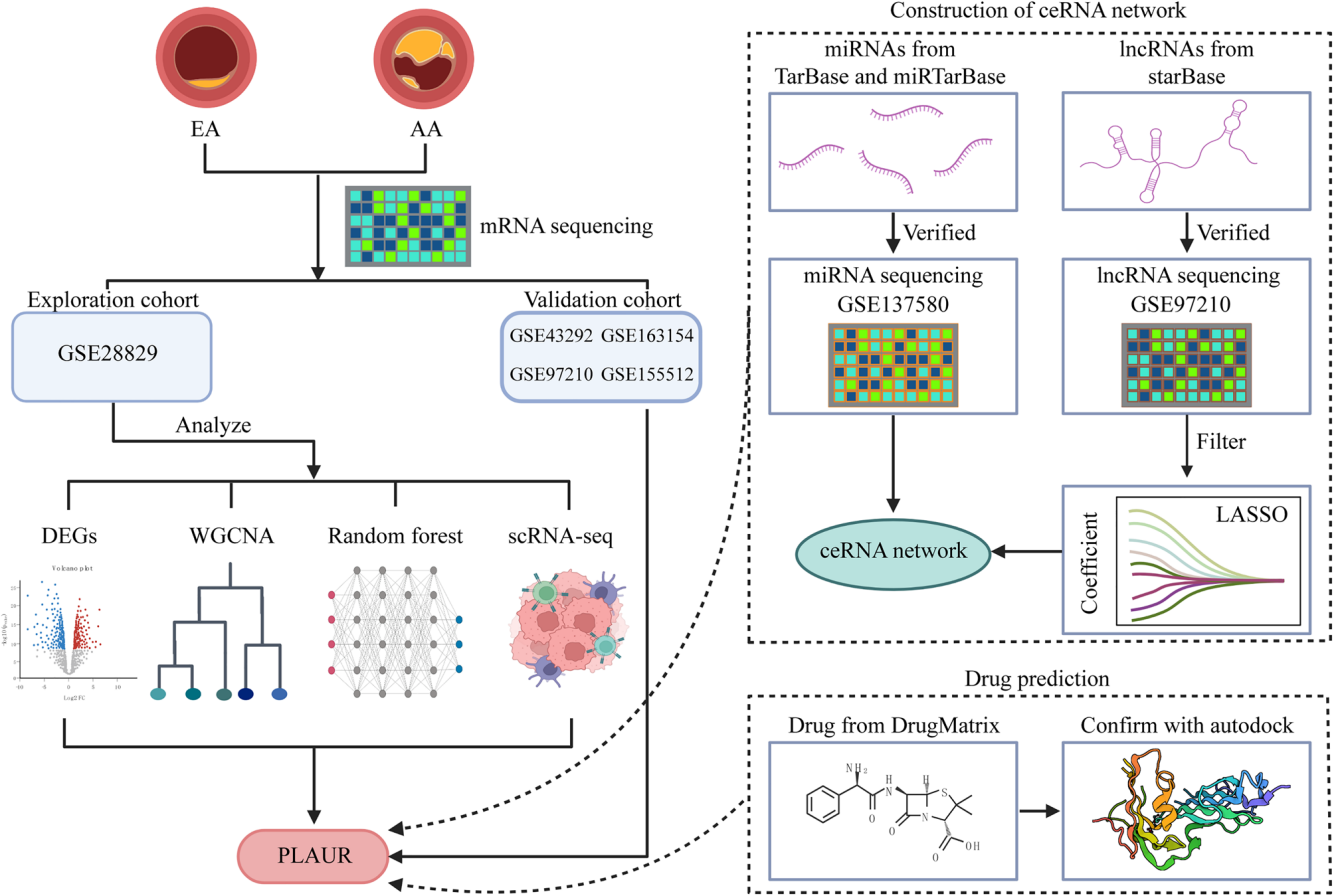
The workflow of this study.

The flowchart of this study is shown in Fig. 1. From Gene Expression Omnibus (GEO; https://www.ncbi.nlm.nih.gov/geo/), the raw gene expression profile for AS was retrieved. The GSE28829 dataset contains 13 early-stage plaques (EA) and 16 advanced-stage plaques (AA) from human carotid artery segments, used as the exploration cohort^12^. Dataset GSE43292 consisting of 32 EA and 32 AA samples used as the validation cohort^13^. Dataset GSE163154 containing 27 plaques with intraplaque hemorrhage (IPH) and 16 plaques without intraplaque hemorrhage (non-IPH) conducted from carotid endarterectomy patients^14^. Dataset GSE97210 characterized lncRNA and mRNA profiles in 3 normal intimal tissues (NA) and 3 AA samples^15^. Dataset GSE137580 analyzed global miRNAs in an AS model of human aortic endothelial cells treated with oxidized LDL. Dataset GSE155512 recorded single-cell data of AS plaques from 1 patient with symptomatic and 2 patients with asymptomatic^16^. ScRNA-seq of 3 cases of AS core (AC) plaque and 3 cases of patient-matched proximal adjacent (PA) portion of the carotid artery in GSE159677^17^. scRNA-seq of 8 cases of AS plaques in GSE131778^18^. Dataset GSE137581 has a total of 6 mRNA samples from mouse vessels divided into two equally sized groups, the high fat diet fed Ldlr−/− group and the wild-type group (Wt). Dataset GSE69187 consisting of 3 high fat diet fed Ldlr−/− mice samples and 3 Wt samples^19^. Dataset GSE72248 consisting of 5 high fat diet fed ApoE−/− mice samples and 5 Wt samples^20^. Dataset GSE76812 consisting of 3 high fat diet fed ApoE−/− mice samples and 3 normal chow diet ApoE−/− mice samples^21^. Total aortae of ApoE−/− mice (n = 3) and Wt mice (n = 3) were removed at the age of 78 weeks and microarrays were prepared from extracts of AS lesions and adventitiae obtained by the use of a laser dissection microscope. The sequencing data are stored in GSE10000^22^.

### Identification of DEGs and functional enrichment analysis

Due to good quality control of the data by publishers, we found that the median gene expression was located at essentially the same level in most samples and that the expression ranges did not differ significantly, indicating that no significant batch effects were found in datasets. For dataset GSE10000 we removed the batch effect using the normalizeBetweenArrays function of the R package “Limma” (S1A). Principal component analysis (PCA) was used to visualize the differences between groups using the R package “pca3d” and to calculate the percentage of variance explained using the R package “factoextra”. Subsequently, the R package “Limma” was applied to identify DEGs^23^. We used *p* < 0.05 and |log2FC|> 0.5 as criteria to select DEGs. We set a low threshold to avoid filtering out biological information and include a broader set of genes for subsequent studies. In addition, given that the specificity of AS samples points to many lncRNAs in GSE97210, we used the more stringent criteria of *p* < 0.05 and |log2FC|> 2 to screen for differentially expressed lncRNAs. We visualized these results with volcano plots and heatmaps using the R package “ggplot2”. We used the “clusterProfiler” package of the R language to perform Gene Ontology (GO) annotation analysis to understand biological processes related to DEGs^24^, and adjust *p* < 0.05 was considered statistically significant. To distinguish biological processes between different subgroups, we implemented the “clusterProfiler” package in R software to conduct gene set enrichment analysis (GSEA) on the gene expression profile dataset. The genes were sequenced in descending order according to log2FC, and annotated gene sets were obtained from the GO database and the Kyoto Encyclopedia of Genes and Genome (KEGG) database, respectively. The screening condition was adjusted *p* < 0.05^25^.

### Construction of WGCNA and identification of hypoxia-related module

We constructed a gene co-expression network based on gene expression profiles from the GSE28829 and GSE43292 datasets using the R package “WGCNA”^26^. Calculated β from 1 to 20 to choose an appropriate soft threshold to convert the adjacency matrix to a topological overlap matrix to build the network. We further divided the initial modules by dynamic tree cutting. We merged similar modules following a height cutoff of 0.15, and genes that were unable to be merged were grouped into gray modules. Finally, the correlation between modules and traits was obtained by Pearson’s correlation analysis. Modules with the most significant correlation with clinical characteristics were selected for follow-up studies. If HIF1A is located in the core region of a module, this module is considered a “hypoxia-related module”, and the genes contained in this module are submitted for GO enrichment analysis.

### Gene set variation analysis (GSVA)

GSVA is a nonparametric and unsupervised enrichment algorithm that estimates changes in specific gene sets based on expression profile data^27^. GSVA scores for each gene set were obtained using the GSVA software package to quantify marker pathway activity. The differences in biological pathways among different subtypes of AS were compared using the R package “Limma”. In this section, pathways with *p* < 0.05 and t > 2 were considered significantly enriched.

### Assessment of immune cell infiltration

The “GSVA” R package was used for the single sample gene set enrichment analysis (ssGSEA) to calculate the abundance of 28 immune cell types in each sample based on the expression levels of specific immune cell marker genes. The set of genes for marking 28 types of immune cells was obtained from a previously published article^28^. The CIBERSORT algorithm (https://cibersort.stanford.edu/) using RNA-sequencing data to estimate the abundance of various types of immune cells in a mixed cell population. The file that records the leukocyte gene signature matrix composed of 547 genes, named LM22, is used by the CIBERSORT algorithm to distinguish 22 immune cell types. We calculated the 22 immune cell types and 4 aggregated immune cell types in the plaques inferred by the CIBERSORT algorithm. The 4 categories of immune cells, namely, total macrophages (sum of the percentages of M0, M1 and M2 macrophages), total mast cells (sum of the percentages of activated and resting mast cells), total lymphocytes (sum of the percentages of B cell naïve, B cell memory, plasma cell, T cells CD8, T cells CD4 naïve, T cells CD4 memory resting, T cells CD4 memory activated, T cells follicular helper, T cells regulatory, T cells gamma delta, NK cells resting, NK cells activated, Monocytes) and total dendritic cells (sum of the dendritic epithelial cells resting and dendritic cells activated). The immune infiltration (ImmuneScore) of the samples was obtained by calculating the immune purity of the expression matrix through the estimation algorithm using the “Estimated” R pacage.

### Identification of hypoxia status in individual samples

Unified Manifold Approximation and Projection (UMAP), a non-linear dimensionality reduction method, can identify the global structure of data according to given features, and subsequently assign a group of patients to different clusters. The set of hallmark genes for hypoxia includes 200 genes was retrieved and obtained from the Molecular Signatures Database (MsigDB; http://www.gsea-msigdb.org/gsea/msigdb/index.jsp) to determine the degree of hypoxia in the samples. After analyzing the clustering results, we determined two groups: “low-hypoxia” and “high-hypoxia”.

### Random forest analysis

The hypoxia gene set is from the “HALLMARK OF HYPOIXA” downloaded from the GSEA website. Then, the GSE28829 data set was randomly divided into a training set (n = 15, EA = 7, AA = 8) and a test set (n = 14, EA = 6, AA = 8). A classifier was constructed using the randomForest function of the R package “randomForest”. The number of better binomial tree variables was selected by looping on the training set. Simultaneous training revealed that the error within the model was largely stable when the number of decision trees contained in the random forest was around 500. So finally a random forest was constructed with ntry = 108 and mytree = 500 on the training set with the hypoxic gene set as the input variable. The classifier is based on the gene expression matrix, and features are compared and ranked by importance. We selected the top 30 genes with the highest average reduction accuracy as candidate genes for subsequent studies. Then, the excellent classification ability of the model was verified on the test set. The classification ability was characterized by Receiver Operating Characteristic (ROC) Evaluation. The ROC curve was also used to evaluate the diagnostic ability of the genes.The ROC contains the area under the curve (AUC) and 95% confidence interval (CI). The genes with AUC > 0.8 were considered to have desirable diagnostic value in this study.

### Analysis of single-cell RNA-sequencing (scRNA-seq) data

We used the R package “Seurat” to analyze single-cell data. The R package Seurat was used to perform downstream analyses. For the GSE15512 and GSE159677 datasets, we retained high quality cells by screening criteria of > 200 unique molecular identifiers (UMIs) and mitochondrial-derived UMI counts below 10%. CAA was then applied to correct batch effects across experimental batches. For the GSE131778 dataset we used the processed matrix provided by the authors. We then constructed a shared nearest neighbor (SNN) graph using 50 nearest neighbors and 20 principal component dimensions as input, followed by a resolution parameter of 0.5 to identify clusters, and visualized these clusters using the unified flowform UMAP. We manually annotated the cell types of each cluster on the basis of some known markers. We used the R package “AUCell” to score the activity of selected pathways of the individual cells, and used density maps to show the activity level of the target pathways in each cluster^29^.

### Competitive endogenous RNA (ceRNA) network construction

First, cross-validation results based on multiple databases predicted that the hypoxia-critical gene HIF1A may be a transcription factor for PLAUR. The data mining platform ChIP-Atlas (http://chip-atlas.org/) and Gene Transcription Regulatory Database (GTRD, http://gtrd20-06.biouml.org/) were used to predict the potential target genes of HIF1A. HumanTFDB is evaluated to be one of the most comprehensive genome-wide transcription factor databases (http://bioinfo.life.hust.edu.cn/HumanTFDB/#!/). JASPAR is a high-quality transcription factor binding profile database (https://jaspar.genereg.net/). We combined the HumanTFDB, GTRD and JASPAR databases to explore whether HIF1A is one of the transcription factors for PLAUR. Next, each node of the ceRNA network that regulates HIF1A was screened based on database prediction and validation of sequencing results. We selected 2 miRNA prediction databases, TarBase (https://carolina.imis.athena-innovation.gr/diana_tools) and miRTarBase (https://mirtarbase.cuhk.edu.cn/) to predict the miRNAs regulating HIF1A. The miRNAs in the overlap of the predicted results from the above database were used for subsequent validation. GSE137580 was used to verify the negative correlation between miRNA and HIF1A. We then cross-validated the candidate miRNAs on 2 independent databases, miRWalk (http://www.ma.uni-heidelberg.de/apps/zmf/mirwalk/) and miRPathDB (https://mpd.bioinf.uni-sb.de/overview.html), to improve the reliability of the predictions. Subsequently, we selected starBase (https://starbase.sysu.edu.cn/) to predict lncRNAs regulating candidate miRNAs according to stringent prediction criteria of the presence of validated evidence in 3 or more CLIP data. GSE97210 was used to verify the positive correlation between candidate miRNA and lncRNA. The least absolute shrinkage and selection operator (LASSO) is a dimension reduction approach that has demonstrated superiority over regression analysis in evaluating high-dimensional data. We used the LASSO algorithm to filter out the candidate lncRNAs from above that are most closely associated with AS progression. We constructed a LASSO regression prediction model via the R package “glmnet” to select the signature lncRNAs most associated with plaque development. The parameters used in the LASSO analysis were α = 1 and nlambda = 100. Choose lambda.min as the optimal lambda^30^.

#### Prediction and validation of potential drugs to slow plaque progression

The Enrichr database (http://amp.pharm.mssm.edu/Enrichr/) was searched using DrugMatrix analysis to identify potential targeted drugs. Furthermore, adjust *p* < 0.05 was set as the screening standard. Autodock is an algorithm-based software application for predicting interactions between small molecule ligands and macromolecular receptors, facilitating computational algorithm-based drug construction, discovery, and virtual screening^31^. To predict the binding conformation and binding free energy of drugs and proteins, virtual tests are performed sequentially in the following three steps: first, the RCSB PDB (https://www.rcsb.org/) was used to obtain the 3D protein structures of target genes; second, the 3D crystal structures of the drugs were acquired from the PubChem database (https://pubchem.ncbi.nlm.nih.gov/) as ligands; finally, Autodock version 4.2.6 was used to predict protein–ligand interactions; in addition, Pymol version 4.6.0 was used to visualize the docking pocket of the ligand-bound target protein with the best affinity. Docking pockets with binding energies below − 2 kcal/mol are considered ideal binding conformations.

## Results

### Hypoxia was identified as a characteristic of AA

**Figure 2:**
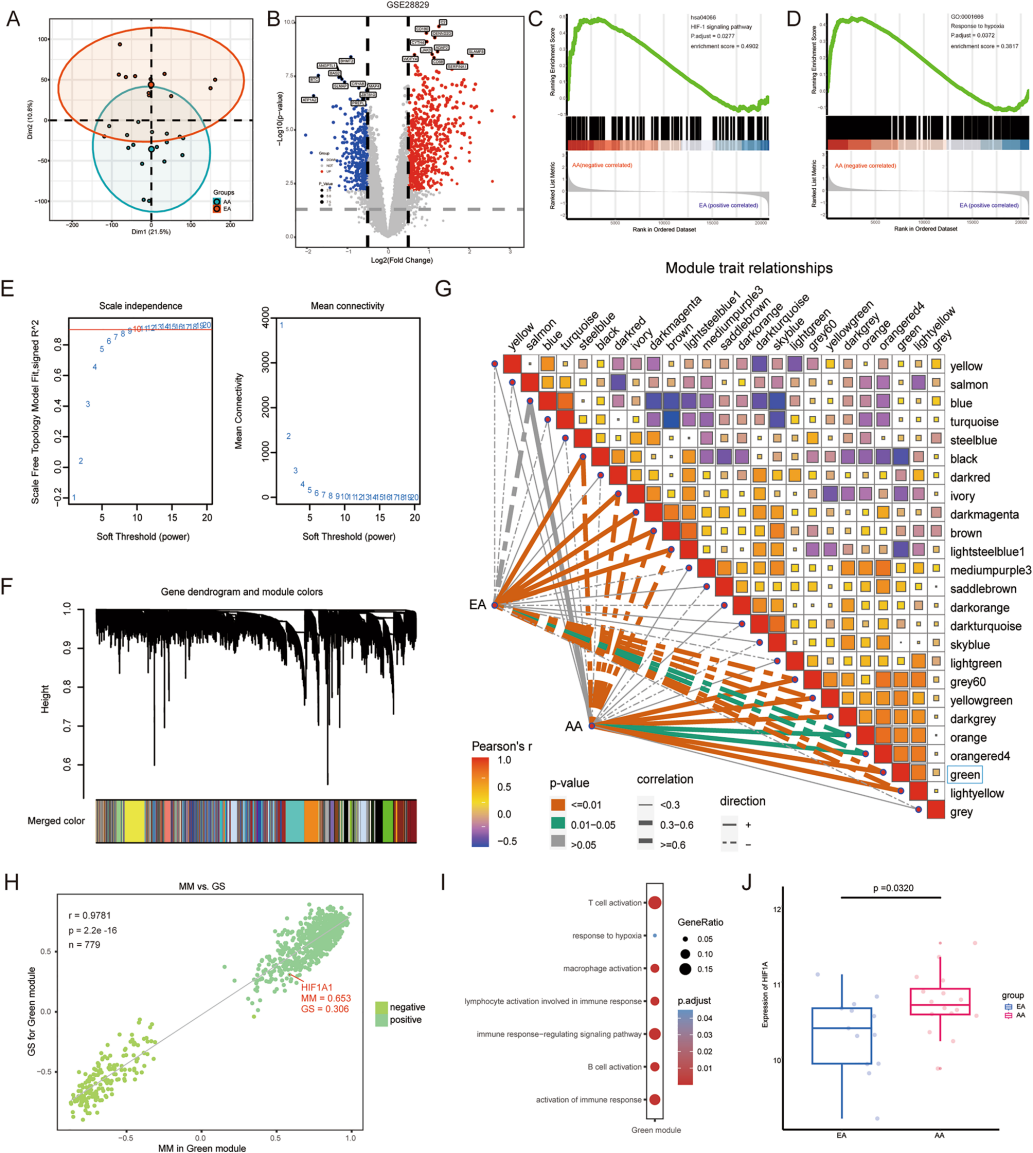
Hypoxia was identified as a characteristic of AA. (**A**) EA and AA were clustered into two clusters with significant heterogeneity in the results of PCA analysis. (**B**) Volcano plot showing the DEGs between EA and AA in the GSE28829 cohort. |Log2FC|> 0.5 and *p* < 0.05 are considered to have a significant statistical difference. Upregualted (red) and downregulated (blue) are indicated. (**C**) GSEA of 107 genes represents HIF-1 signal pathway, which reveals the relationship between plaque status and the HIF-1 pathway. AA is located on the left side near the starting point of the x-axis, while EA is located on the right side of the x-axis. (**D**) GSEA of 291 marker genes represents “hypoxia response”, illustrating the relationship between plaque status and hypoxia response. AA is located on the left side near the origin of the x-axis, while EA is located on the right side of the x-axis. (**E**) Soft threshold selection process. (**F**) Cluster dendrogram. Each color represents one specific co-expression module and the colored rows below the dendrogram represent the merged modules. (**G**) Heatmap showing the relationship between different modules and the correlation between modules and plaque status. A large square area indicates a high correlation between modules, a deep color indicates a small p value. Positive (red) and negative (blue) are indicated. Correlation between the module and the status is indicated by the thickness of the line. Different colors indicate different p value intervals. Positive (solid line) and negative (dotted line) are indicated. The green module exhibited the highest correlation with hypoxia (r = 0.8000, *p* = 2.0e−07) and was considered a “hypoxia-related module”. (**H**) The module membership and gene significance of 779 genes involved in the green module exhibited a highly positive correlation (r = 0.9781, *p* = 2.2e−16), and HIF1A was located in the core part which is positively correlated with hypoxia. (**I**) For the GO functional annotation of the 779 genes included in the green module, seven representative statistically significant items were selected. (**J**) HIF1A was significantly increased in AA (*p* = 0.0320).

The reduction and visualization of transcriptome data from the exploration cohort, GSE28829, using the PCA algorithm showed significant heterogeneity between EA and AA (Fig. 2A). With a threshold of *p* < 0.05 and |log2FC|> 0.5, 625 highly elevated genes and 374 significantly downregulated genes were discovered in AA samples of the exploration cohort (Fig. 2B). The HIF-1 signaling pathway genes were then significantly enriched in the AA samples by GSEA analysis as shown in Fig. 2C (enrichment score = 0.4902, adjust *p* = 0.0277). Similarly, GSEA analysis found a significant enrichment of genes related to the biological process of the “hypoxia response” in AA samples, as shown in Fig. 2D (enrichment score = 0.4902, adjust *p* = 0.0267). WGCNA constructed a scale-free co-expression network using all transcriptome data and plaque pathologic status of the GSE28829 dataset. We set the soft threshold at 10 to build the scale-free network. At that time, the fit index was > 0.85 and the average connectivity was close to 0 (Fig. 2E). Subsequently, dynamic tree cutting was used to generate co-expression modules, and a total of 25 modules were generated in the co-expression network (Fig. 2F). The graph of the relationship between the modules and their corresponding clinical features showed that the green module revealed the strongest correlation (module-trait weighted correlation = 0.8016, *p* = 1.7e−07) with AA and was considered the key module for plaque development (Fig. 2G). The green module contains 779 genes, which are highly positively correlated in terms of module members and gene significance (r = 0.9781, *p* = 2.2e−16). It should be noted that HIF1A, the molecular markers of hypoxia, is located in the core of the green module (Fig. 2H). GO function enrichment analysis showed that genes contained in the green module participated mainly in the response to hypoxia (Fig. 2I). In addition, we noted that GO functional enrichment revealed that hypoxia-related genes were also widely involved in immune-related biological processes, including T cell activation, macrophage activation, B cell activation, etc. The boxplot indicated that HIF1A expression was much higher in AA compared to EA (*p* = 0.0320; Fig. 2J). Based on the above information, we defined the green module as a “hypoxia-related module”, and identified 779 of the genes as hypoxia-related genes, which promote plaque formation by participating in response to hypoxia.

### The hypoxia microenvironment is related to the immunological characteristics of the plaques

**Figure 3:**
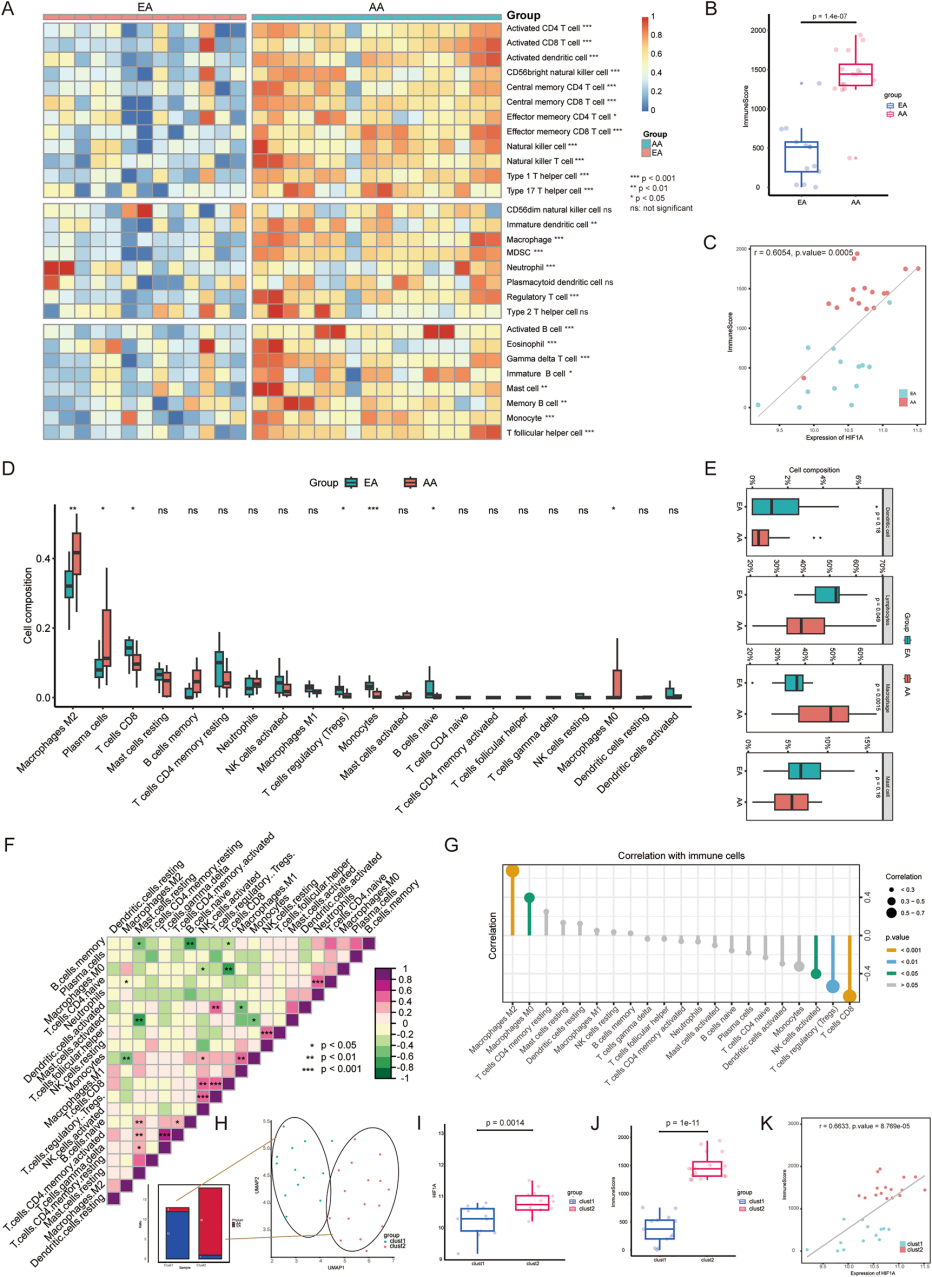
Microenvironment of hypoxia is related to immunological characteristics of plaques. (**A**) Heatmap showing the relationship between the infiltrated proportion of 24 immune cells and plaque status (EA and AA) in the GSE28829 cohort, based on the ssGSEA algorithm (*p* < 0.05*; *p* < 0.01**; *p* < 0.001***; ns = not significant). (**B**) ImmuneScore was significantly increased in AA (*p* = 1.4e−07). (**C**) Correlation between HIF1A and ImmuneScore (r = 0.6054, *p* = 0.0005). (**D**) Boxplots showing differences in the abundance of different immune cell infiltrates between EA and AA. (*p* < 0.05*; *p* < 0.01**; *p* < 0.001***; ns = not significant). (**E**) Boxplots showing the difference between EA and AA in the infiltration abundance of four categories of immune cells calculated based on CIBERSORT results. (**F**) Heatmap showing the relationship between 21 kinds of immune cells. Positive (purple) and negative (green) are indicated, with darker color indicating stronger relationship. (*p* < 0.05*; *p* < 0.01**; *p* < 0.001***; blank means not significant). (**G**) Correlation between HIF1A and immune cell abundance based on CIBERSORT score. (**H**) The GSE28829 cohort was clustered into cluster1 and cluster2 by UMAP based on marker gene set of hypoxia and the lower left part shows the specific allocation of EA and AA in cluster1 and cluster2. (**I**) The transcription level of HIF1A was significantly increased in clust2 (*p* = 0.0014). (**J**) ImmuneScore was significantly increased in cluster2 (*p* = 1.0e−11). (**K**) Correlation between HIF1A and ImmuneScore (r = 0.6633, *p* = 9.7e−05).

The immunological characteristics of the plaques were evaluated according to immune cell infiltration and ImmuneScore. The invasion of 28 different types of immune cells was estimated using ssGSEA. The heatmap revealed that most of the innate and adaptive immune cells except CD56dim natural killer cells, plasma cell like dendritic cells and type 2 T helper cells showed higher level of infiltration in AA (Fig. 3A). Compared to EA, ImmuneScore in AA increased dramatically, according to the boxplot (*p* = 1.4e−07; Fig. 3B). The scatter plot showed that there was a significant positive correlation between HIF1A and ImmuneScore (r = 0.6054, *p* = 0.0005; Fig. 3C). Then we used CIBERSORT to evaluate the infiltration of 22 immune cell types in plaques. The boxplots showed that compared to EA, the infiltration of Macrophages M2, Macrophages M0, Plasma cells rested T cells, and NK cells were significantly increased in AA, while the infiltration of T cells CD8, T cells regulatory (Tregs), Monocytes and B cells naïve were significantly reduced (Fig. 3D). Boxplots showed that only total macrophages were significantly increased in abundance among the 4 aggregated immune cell types in AA, but total lymphocyte infiltration was significantly decreased (Fig. 3E). Additionally, the results of the correlation study revealed a strong association between several immune cells. Heatmap showed that macrophage M0 was significantly negatively correlated with NK cells activated and T cells CD8, and macrophage M1 was significantly negatively correlated Dendritic cells activated but significantly positively correlated with monocytes, and macrophage M2 was significantly negatively correlated with T cells CD4 naïve and monocytes (Fig. 3F). The results of the correlation analysis showed that HIF1A expression was positively correlated with the abundance of macrophage M2 and macrophage M0, but significantly negatively with the infiltration abundance of NK cell activation, T cell regulation (Tregs) and T cell CD8 (Fig. 3G). Using 200 hypoxia marker genes from MSigDB, each sample of GSE28829 cohort was allocated to the nearest cluster based on UMAP algorithm, and two clusters, cluster 1 and cluster 2, were obtained (Fig. 3H). Cluster 1 and cluster 2 contained 13 and 16 patients, respectively. Only one AA sample was assigned to cluster 1, and only one EA sample was assigned to cluster 2 (Fig. 3H). Comparing clusters 1 and 2, the boxplot revealed that cluster 2 had a much higher expression of HIF1A (*p* = 0.0014; Fig. 3I). Therefore, cluster 1 and cluster 2 are defined as “low-hypoxia cluster” and “high-hypoxia cluster” respectively. The clustering results again showed that hypoxia was an important characteristic of AA. There was a significant increase in ImmuneScore of the low-hypoxia cluster compared to high-hypoxia cluster (*p* < 1.0e−11; Fig. 3J). The scatter plot shows that there is a significant positive correlation between HIF1A and ImmuneScore in the defined clusters (r = 0.6633, *p* = 8.7e−05; Figure Fig. 3K). These results suggest that hypoxia may affect the number and phenotype of immune cells infiltrating the plaque by overexpressing HIF1A, thus mediating the progression of plaque.

### Identification of AA-specific candidate genes involved in hypoxia

**Figure 4:**
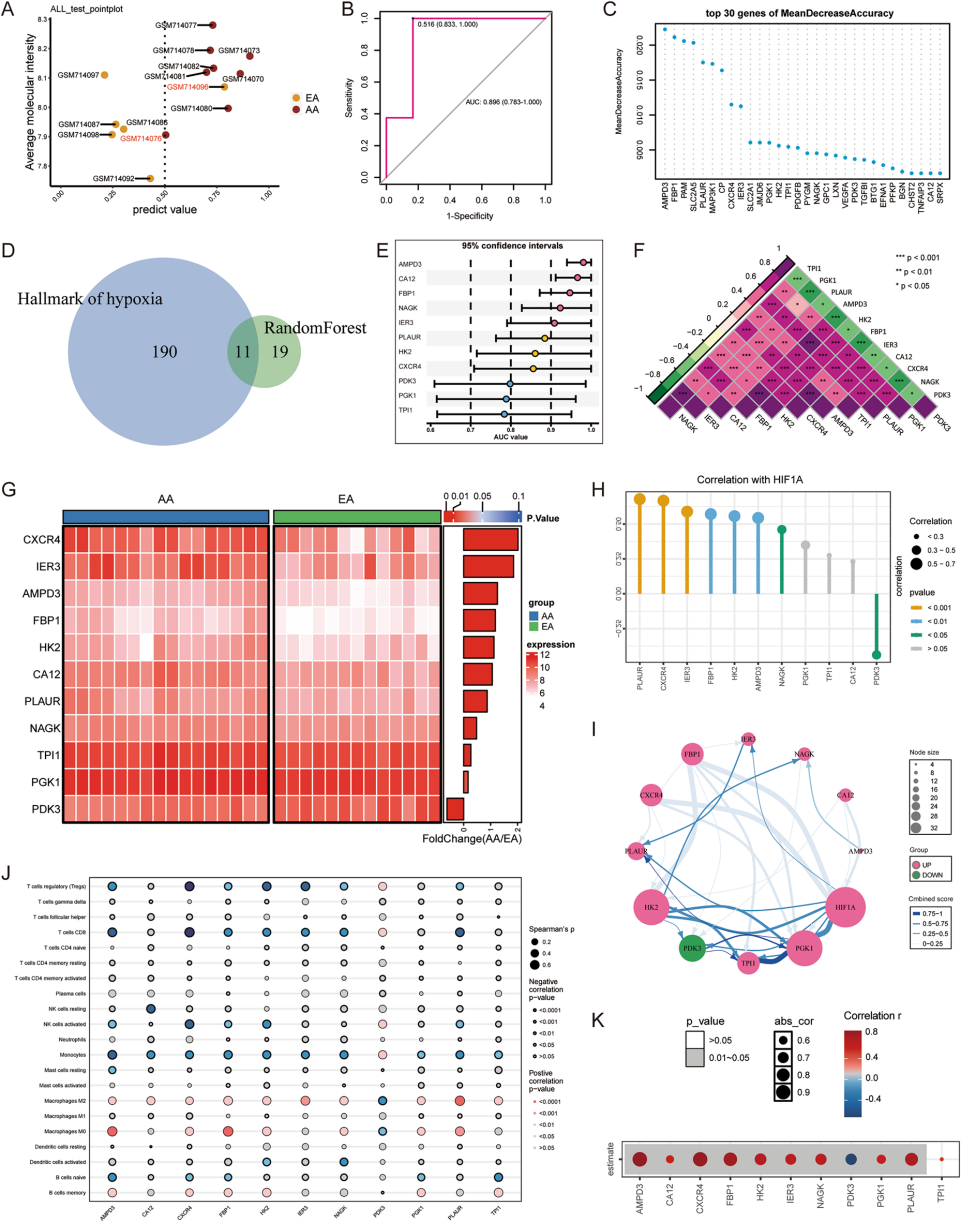
Identification of AA-specific candidate genes involved in hypoxia. (**A**) Performance of the model in the training cohort. (**B**) ROC of the random forest model in the test cohort, AUC = 0.896 (95% CI 0.783–1.000). (**C**) Top 30 genes prioritized by random forest analysis ranked by the mean decrease in accuracy. (**D**) Venn plot showing a total of 11 important variables that overlap between the “Hallmark of hypoxia” gene set and the genes in the “hypoixa-related module”. (**E**) Dumbbell plot showing the AUC values and 95% confidence intervals of each candidate gene. (**F**) Heatmap showing the correlation between candidate genes. (**G**) Heatmap showing differential expression of candidate genes between EA and AA. (**H**) Lollipop plot showing the correlation between HIF1A and candidate genes. The size of the ball indicates the magnitude of the correlation and different colors indicate different p values. (**I**) A correlation network involving the 11 candidate genes and HIF1A in the GSE28829 cohort. (**J**) Spearman correlation between candidate genes and 21 immune cells. The size of the bubble represents the correlation level. The color of the bubble represents p. Red: positive correlation. Blue: negative correlation. (**K**) Correlation between candidate genes and ImmuneScore. Positive (red) and negative (blue) are indicated. Grey mask represents *p* < 0.05 and white mask represents *p* > 0.05.

We used the random Forest algorithm to further identify which hypoxia-related genes in the “hypoxia-related module” are closely associated with plaque progression. A training group (7 cases EA, 8 cases AA) and a test group (6 cases EA, 8 cases AA) were randomly assigned from the GSE28829 cohort. We built a random forest machine learning model based on the transcriptome data of the training group, which showed good recognition ability in the test set, with only 1 case of EA (GSM714096) and 1 case of AA(GSM714076) not being correctly identified (Fig. 4A). The model achieved an AUC of 0.896 in the test group (Fig. 4B). Figure 4C shows the prioritization of the first 30 important variables ranked according to the average reduced accuracy. The Venn diagram showed that a total of 11 important variables overlapped with the “Hallmark of hypoxia” gene set published in the MsigDB database and were selected as candidate genes in our analysis that followed (Fig. 4D). The 11 candidate genes including AMPD3, CA12, FBP1, NAGK, IER3, PLAUR, HK2, CXCR4, PDK3, PGK1, TPI1. The dumbbell plot showed the AUC values and 95% confidence intervals under the ROC curve for each candidate gene to evaluate their diagnostic performance in predicting plaque progression in the GSE28829 cohort (Fig. 4E). Candidate genes other than PDK3, PGK1, and TPI1 have a high diagnostic value for AA (AUC > 0.8), which means that these genes have important contributions to the development of AS.

The correlation analysis of these 11 candidate genes revealed significant negative co-expression between PDK3 and other genes. In addition, all genes except PDK3 have a significant positive correlation, suggesting that they have a co expression relationship (Fig. 4F). Using |log2FC|> 0.5, *p* < 0.05 as a threshold for differential expression analysis, the results indicated that the transcript level of CXCR4, IER3, AMPD3, FBP1, HK2, CA12 and PLAUR in AA increased significantly, while the expression of PDK3 decreased significantly (Fig. 4G). Significant differences in expression suggest that these candidate genes are involved in the development of AS. Then, we explored the relationship between these 11 candidate genes and HIF1A, the molecular feature of hypoxia response. The results of the Pearson’s correlation test showed that HIF1A was significantly positively correlated with PLAUR, CXCR4, IER3, FBP1, HK2, AMPD3, and NAGK and negatively correlated with PDK3 (Fig. 4H). The protein interaction network between the 11 candidate genes and HIF1A involved in the exploration cohort based on the analysis of the STING database was shown in Fig. 4I. Since the hypoxia microenvironment may be involved in shaping the immune characteristics of plaques, we further explored the potential association between 11 candidate genes and infiltration of 22 immune cell types (Fig. 4J). In addition, the results of the correlation analysis between the candidate genes and ImmuneScore showed that TPI1 had no significant correlation with ImmuneScore, PDK3 had a significant negative correlation with ImmuneScore, and other genes had a significant positive correlation with ImmuneScore (Fig. 4K). In conclusion, we preliminarily selected 11 Hypoxia-related genes that were highly associated with AS. The effect of these genes on AS may be mediated in part by affecting immune infiltration. In conclusion, we preliminarily screened 11 candidate genes, some of which can recognize AA well, indicating that they are highly related to plaque progression, which may be mediated by regulating immune cell infiltration.

### Identification of PLAUR as the most valuable AA-specific candidate gene involved in hypoxia

Subsequently, we verified the robustness of the appeal candidate genes. First, we verified whether the independent dataset GSE43292 is suitable as the validation cohort. Reduction and visualization of transcriptome data from the validation cohort using the PCA algorithm showed significant heterogeneity between EA and AA (S1B). With a threshold of *p* < 0.05 and |log2FC|> 0.5, 650 significantly upregulated genes and 510 significantly downregulated genes were found in AA samples of the validation cohort (S1C). Then, the HIF-1 signaling pathway genes were significantly enriched in AA samples by GSEA analysis, as shown in S1D (enrichment score = 0.4819, adjust *p* = 0.0084). Similarly, the GSEA analysis found a significant enrichment of genes related to the biological process of the “hypoxia response” in AA samples, as shown in S1E (enrichment score = 0.4902, adjust *p* = 0.0345). The boxplot showed that HIF1A increased significantly in AA compared to EA (*p* = 0.0002; S1F). The boxplot showed that ImmuneScore was significantly increased in AA compared to EA (*p* = 2.7e−06; S1G). The scatter plot showed that there was a significant positive correlation between HIF1A and ImmuneScore (r = 0.7951, *p* = 4.2e−15; S1H). These results showed that GSE43292 and GSE28829 have similar internal characteristics, which means that it is very appropriate to use the GSE4392 dataset as the validation cohort.

**Figure 5:**
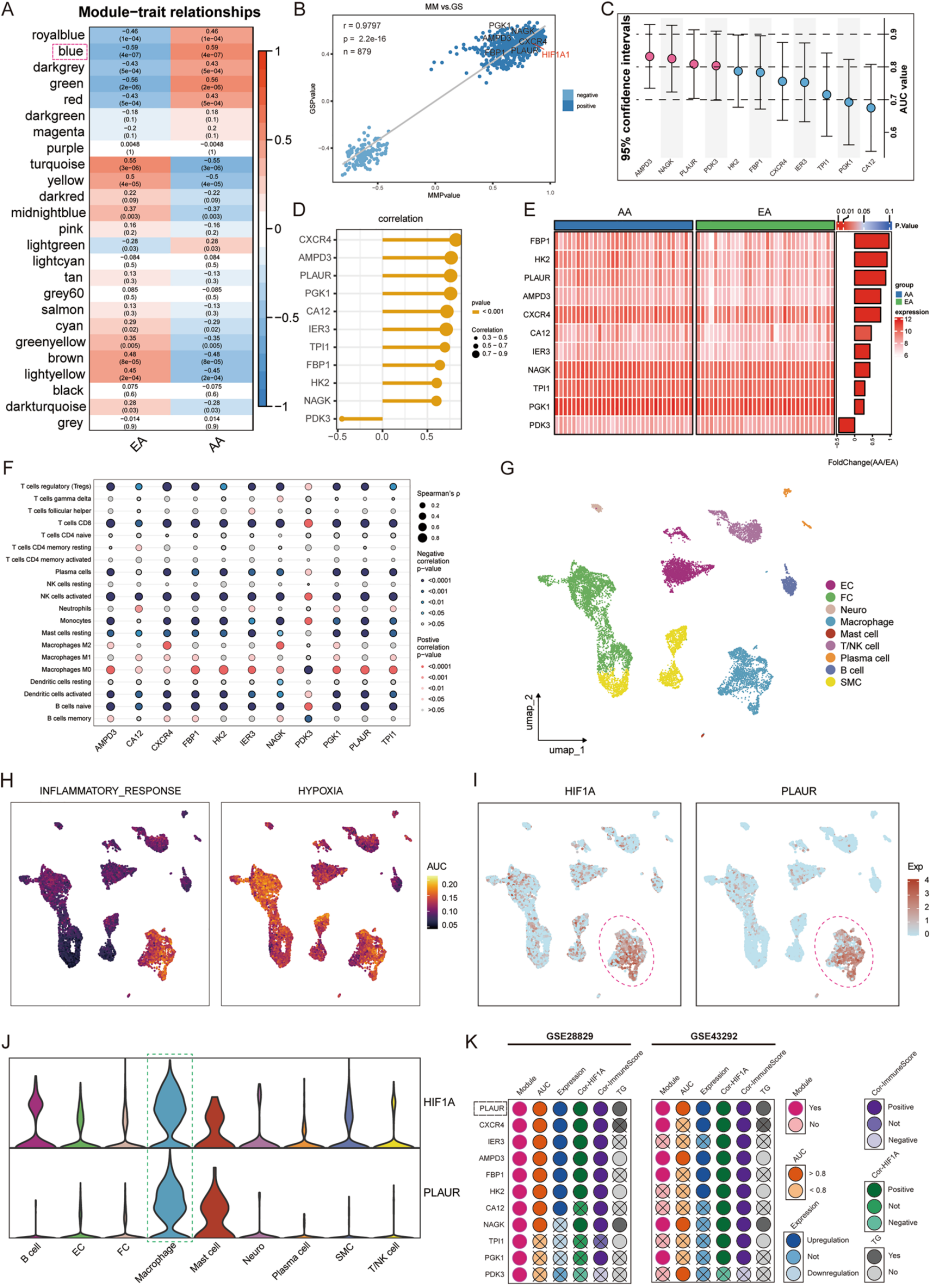
Validation and screening of AA-specific candidate genes involved in hypoxia. (**A**) Heatmap of the correlation between module eigengens and the occurrence of SLE. (**B**) Correlation between module membership of the Blue module and gene significance (r = 0.9797, *p* = 2.2e−16). (**C**) Dumbbell plots show the AUC values and 95% confidence intervals of each candidate gene. (**D**) Lollipop plot showing the correlation between HIF1A and candidate genes. The size of the ball indicates the magnitude of the correlation and different colors indicate different p values. (**E**) Heatmap showing differential expression of candidate genes between EA and AA. (**F**) Spearman correlation between transcription levels of characteristic genes and 21 immune cells. The size of the bubble represents the correlation level. The color of the bubble represents p values. Red: positive correlation. Blue: negative correlation. (**G**) Further annotated to 10 cell types based on known markers, including EC, FC, SMC, Neuro, B cells, T cells, NK cells, Plasma cell, Mast cell, and Macrophage. Each dot corresponds to an individual cell, colored according to the cell cluster. EC indicates endothelial cell; FC, fibroblast cell; and SMC, smooth muscle cell. (**H**) Density plot showing AUC scores for individual cells with “INFLAMMATORY RESOPONSE” and “HYPOXIA” pathway activity. (**I**) Density plot showing the expression of HIF1A and PLAUR in different cell types. (**J**) Violin plots showing the expression of HIF1A and PLAUR in each cell types. (**K**) Status of 11 candidate genes in GSE28829 and GSE43292.

Subsequently, a scale-free co-expression network was constructed by WGCNA using all transcriptome data and plaque pathologic status from the GSE43292 dataset. We set the soft threshold as 16 to build the scale-free network. At this time, the fit index was > 0.85 and the average connectivity was close to 0 (S2A). Subsequently, dynamic tree cutting was used to generate co-expression modules and a total of 25 modules were generated in the co-expression network (S2B). The graph of the relationship between modules and their corresponding clinical features showed that the blue module revealed the strongest correlation (module-trait weighted correlation = 0.5874, *p* = 4.1e−07) with AA and was regarded as key module for plaque development (Fig. 5A). The blue module contains 879 genes, which are highly positively correlated in terms of module members and gene significance r = 0.9797, *p* = 2.2e−16) and HIF1A is located in the core part of blue module (Fig. 5B). Therefore, the green module was determined as the “hypoxia-related module” in the GSE43292 cohort, and we noted that PGK1, AMPD3, FBP1, NAGK, CXCR4, and PLAUR are included in this module. The correlation analysis of these 11 candidate genes showed that there was significant negative correlation between PDK3 and other genes. In addition, all genes except PDK3 have a significant positive correlation, suggesting that they have a co-expression relationship (S2C). The dumbbell plot showed the AUC values and the 95% confidence intervals under the ROC curve for each candidate gene to evaluate their diagnostic performance in predicting plaque progression in the GSE43292 cohort (Fig. 5C). Candidate genes other than AMPD3, NAGK, PLAUR and PDK3 have a high diagnostic value for AA (AUC > 0.8), which means that these genes have important contributions to the development of AS. The results of the Pearson’s correlation test showed that HIF1A was significantly positively correlated with CXCR4, AMPD3, PLAUR, PGK1, CA12, IER3, TPI1, FBP1, HK2, and NAGK and negatively correlated with PDK3 (Fig. 5D). Using |log2FC|> 0.5, p < 0.05 as the threshold for differential expression analysis in GSE43292, the results showed that the expression of CXCR4, IER3, AMPD3, FBP1, HK2, CA12 and PLAUR in AA increased significantly (Fig. 5E). Then we explored the potential association between 11 candidate genes and infiltration of 22 immune cell types (Fig. 5F). In addition, PDK3 exhibited a substantial negative association with ImmuneScore, according to the results of the correlation analysis between candidate genes and ImmuneScore, while other genes had a significant positive correlation (S2D).

We then predicted the target genes of transcription factor HIF1A using GTRD and Chip-Atlas databases. It turned out that 5 of the above 11 candidate genes were predicted in both databases, including PLAUR, AMPD3, HK2, NAGK and TPI1 (S2E). To strengthen the reliability of the prediction results above, we performed cross-validation. Based on HumanTFDB, GTRD, and JASPAR databases, we predicted 7, 10, and 10 possible binding sites for HIF1A as a transcription factor regulating PLAUR expression (Supplementary Tables 1, 2, and 3). Subsequently, to further enhance the reliability of transcriptional regulatory relationship prediction results and to generate a comprehensive map of the landscape of hypoxia in human AS lesions at single-cell resolution, we analyzed the GSE131778 dataset. After rigorous quality control (S2F), 11,403 cells were finally retained for downscaling clustering analysis (S2G). After descending clustering, these cells were identified into 9 cell types, endothelial cell (EC), fibroblast cell (FC), smooth muscle cell (SMC), Neuro, T/NK cell, B cell, Plasma cell, Mast cell and Macrophage, respectively (Fig. 5G). This was based on manual annotation of typical cell type markers (S2H). After calculating the pathway score for each cell it was found that the inflammatory response was significantly increased only in the macrophage cluster, suggesting that macrophages may be involved in shaping the immune landscape of AS (Fig. 5H). We also found that hypoxia was remarkably enriched in all clusters except B cells, suggesting a widespread hypoxia environment in AS lesions. HIF1A expression was remarkably higher in the macrophage cluster than in other cell clusters (Fig. 5I,J). Notably, PLAUR was also significantly overexpressed in the macrophage cluster, indicating a potential co-expression relationship between PLAUR and HIF1A (Fig. 5IJ). This is consistent with the prediction that HIF1A may be a potential transcription factor for PLAUR.

Considering the following contents of 11 candidate genes in the exploration cohort and validation cohort, including whether they are contained in the “hypoxia-related module”, expression, prediction ability (AUC value), co-expression relationship with HIF1A, correlation with ImmuneScore, and whether they are target genes of HIF1A, we finally determined that PLAUR is the most robust gene involved in plaque development (Fig. 5K).

### External validation of PLAUR as a valid diagnostic marker of AS progress

We first validated the expression of Hif1a and Plaur in the two most commonly used well-established mouse models of AS. Boxplots showed that the expression of Hif1a and Plaur in mouse arterial tissue was significantly elevated in Ldlr−/− model mice (S3A). Boxplots showed that the expression of Hif1a and Plaur in mouse AS lesions was significantly elevated in ApoE−/− model mice compared to the outer and inner membranes of Wt mice (S3B). Considering the effective diagnostic efficacy of PLAUR in human AS coronary arteries, we sought to determine whether Plaur also has this classification ability in mouse models of AS. Boxplots showed that Plaur expression in mouse arterial tissues was considerably elevated in Ldlr−/− model mice, and, the AUC value reached 1.000 suggesting an excellent predictive ability of Plaur in the GSE69187 cohort (S3C). In another Ldlr−/− mouse cohort, GSE76812, it was observed that the expression of Plaur in mouse AS lesions was significantly higher in model mice fed a high-fat diet compared to normal diet Ldlr−/− mice, suggesting the involvement of Plaur in the exacerbation of AS lesions. Moreover, an AUC value of 1 suggested an excellent ability of Plaur to diagnose lesion progression in the GSE76812 cohort (S3D). We also observed in ApoE−/− model mice that Plaur expression was significantly elevated in arteries with AS lesions. An AUC value of 0.88 indicated an excellent ability of Plaur to classify atherosclerotic lesions in the GSE72248 cohort (S3E). The results of the mouse AS model again support the possibility that Plaur may participate in the progression of AS and has outstanding diagnostic capabilities for lesions.

**Figure 6:**
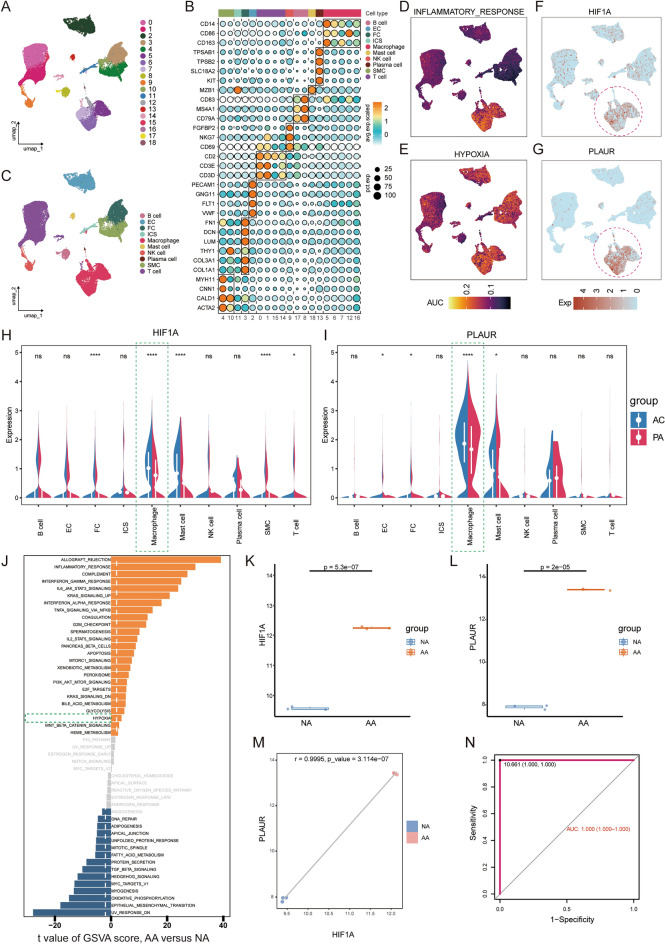
External validation of PLAUR in the progression of AS. (**A**) UMAP projections of 46,347 single cells from the AC in 3 cases and the PA in 3 matched cases were shown to form 19 major clusters. (**B**) Dot plots showing the average expression of known markers in the cell types represented. (**C**) Further annotated to 10 cell types based on known markers, including EC, FC, SMC, ICS, B cells, T cells, NK cells, Plasma cell, Mast cell, and Macrophage. Each dot corresponds to an individual cell, colored according to the cell cluster. ICS indicates an intermediate cell state. (**D**, **E**) Density plot showing AUC scores for individual cells with “INFLAMMATORY RESOPONSE” and “HYPOXIA” pathway activity. (**F**, **G**) Density plot showing the expression of HIF1A and PLAUR in different cell types. (**H**, **I**) Violin plots showing the expression of HIF1A and PLAUR in each cell types in different pathological states and the significance of the differences. (**J**) Differences in pathway activities scored per samples by GSVA between AA and NA. Bar graphs show t-values from linear models, t > 2 are considered significant different. Orange indicates pathways enriched in AA, blue indicates pathways enriched in NA. (**K**) HIF1A was significantly increased in AA (*p* = 5.3e−07). (**L**) PLAUR was significantly increased in AA (*p* = 2.0e−05). (**M**) Correlation between HIF1A and PLAUR (r = 0.9995, *p* = 3.1e−07). (**N**) ROC curves of PLAUR in GSE97210 dataset, AUC = 1.000 (95% CI 1.000–1.000).

To further validate that PLAUR plays a crucial role in the progression of AS plaques, while complementing the non-direct evidence of HIF1A as a potential transcription factor for PLAUR, we explored it on two additional independent human datasets. For the single-cell dataset GSE159677, 46,347 cells were retained after quality control, of which 35,004 were from AC and 11,343 were from PA (S4A and S4B). All samples showed good overlap after removal of batch effects (S4C, S4D). Reduced-dimensional clustering yielded 19 cell clusters (Fig. 6A). Subsequently, manual clustering annotation based on classical cell type markers identified 10 groups of cells, including EC, FC, intermediate cell state (ICS), SMC, B cell, T cell, NK cell, Plasma cell, Mast cell and Macrophage (Fig. 6B,C). Calculation of the pathway scores for each cell revealed that, as in the GSE131778 dataset, only the macrophage cluster had a significantly increased inflammatory response. This result suggests that macrophages are the main cell type involved in shaping the chronic inflammatory environment of AS lesions (Fig. 6D). We also found that, consistent with the findings above, hypoxia was significantly enriched in all clusters except B cell and Plasma cell, suggesting a widespread hypoxia environment in AS lesions (Fig. 6E). Expression of HIF1A was significantly higher in the macrophage cluster than in other clusters (Fig. 6F,H). The significant enrichment and high expression of PLAUR in macrophages again demonstrated a potential co-expression relationship between PLAUR and HIF1A (Fig. 6G,I). This strengthened the prediction that HIF1A may be a potential transcription factor for PLAUR. Considering the critical role of macrophages in the development of AS, we suggested the high expression of PLAUR in macrophages indicates the involvement of PLAUR in the progression of AS (Fig. 6I).

We then validated the diagnostic ability of PLAUR for AS progression in the GSE97210 dataset. First, the detection of GSVA was performed and a direct comparison between AA and NA revealed that hypoxia was one of the enriched signatures in AA (Fig. 6J). The boxplot showed that the expression of HIF1A was substantially higher in AA than in NA (*p* = 5.3e−07; Fig. 6K). Therefore, the GSE97210 dataset is applicable as an external validation cohort. Subsequently, we detected the expression of PLAUR and found that the expression of PLAUR increased significantly in AA compared to NA (*p* = 2.0e−05; Fig. 6L). The scatter plot showed that there was a significant positive correlation between HIF1A and PLAUR (r = 0.9995, *p* = 3.1e−07; Fig. 6M). According to the ROC analysis, PLAUR showed excellent predictive ability in the GSE97210 cohort, and its AUC value reached 1.000 (Fig. 6N). The comprehensive results of GSE28829, GSE43292, and GSE97210 show that PLAUR has a stable and excellent ability to predict plaque development.

### PLAUR may serve as A diagnostic marker for concomitant clinical symptoms of AS.

**Figure 7:**
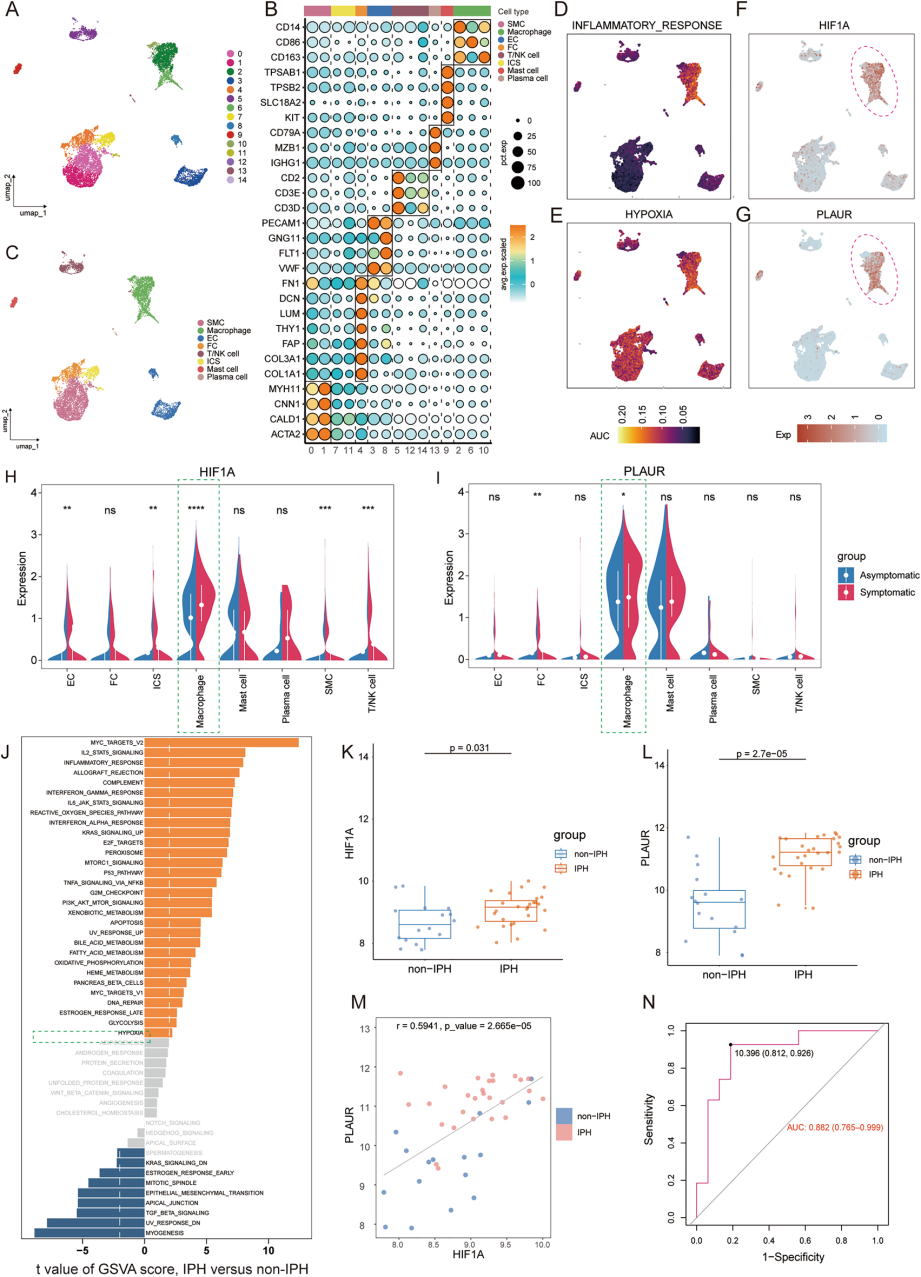
PLAUR May Serve as A Diagnostic Marker for Concomitant Clinical Symptoms of AS. (**A**) UMAP projections of 8867 single cells from the symptomatic in 1 case and the asymptomatic in 2 cases were shown to form 15 major clusters. (**B**) Dot plots showing the average expression of known markers in the cell types represented. (**C**) Further annotated to 8 cell types based on known markers, including EC, FC, SMC, ICS, T/NK cells, Plasma cell, Mast cell, and Macrophage. Each dot corresponds to an individual cell, colored according to the cell cluster. (**D**, **E**) Density plot showing AUC scores for individual cells with “INFLAMMATORY RESOPONSE” and “HYPOXIA” pathway activity. (**F**, **G**) Density plot showing the expression of HIF1A and PLAUR in different cell types. (**H**, **I**) Violin plots showing the expression of HIF1A and PLAUR in each cell types in different pathological states and the significance of the differences. (**J**) Differences in pathway activities scored per samples by GSVA between IPH and non-IPH. Bar graphs show t-values from linear models, t > 2 are considered significant different. Orange indicates pathways enriched in IPH, blue indicates pathways enriched in non-IPH. (**K**) HIF1A was significantly increased in AA (*p* = 5.3e−07). (**L**) PLAUR was significantly increased in AA (*p* = 2.0e−05). (**M**) Correlation between HIF1A and PLAUR (r = 0.5941, *p* = 2.6e−05). (N) ROC curves of PLAUR in GSE163154 dataset, AUC = 0.882 (95% CI 0.765–0.999).

To explore the potential of PLAUR to diagnose concomitant symptoms of AS while complementing the non-direct evidence for HIF1A as a potential transcription factor for PLAUR, we explored it on two additional independent datasets. For the single-cell dataset GSE155512, 8867 cells were retained after quality control, of which 2614 were from a patient with symptomatic and 6253 were from 2 patients with asymptomatic (S4E and S4F). All samples showed an adequate overlap after removal of batch effects (S4G, S4H). Reduced dimensional clustering yielded 15 cell clusters (Fig. 7A). Subsequently, manual clustering annotation based on classical cell type markers identified 8 groups of cells namely EC, FC, ICS, SMC, T/NK cell, Plasma cell, Mast cell and Macrophage (Fig. 7B,C). Consistent with the results above, the results of calculating the pathway score for each cell again demonstrated a significant increase in the inflammatory response of only macrophage clusters, which strengthens the evidence that macrophages are involved in shaping the inflammatory environment of AS (Fig. 7D). We also found that hypoxia was significantly enriched in all clusters except plasma cells. Combining the above results, we conclude that a widespread hypoxia environment is stable present in AS lesions (Fig. 7E). The results showed that HIF1A expression was significantly higher in theMacrophage cluster than in other clusters (Fig. 7F,H). Meanwhile, PLAUR was significantly enriched and overexpressed in the Macrophage cluster (Fig. 7G,I). Combined with the previously mentioned experimental results, we have stable non-direct evidence for a co-expression relationship between HIF1A and PLAUR, which strongly suggests that HIF1A may be a potential transcription factor for PLAUR. Meanwhile, we found that PLAUR expression was significantly higher in macrophage populations in symptomatic individuals, suggesting that PLAUR may be involved in the transition from stable to unstable AS lesions (Fig. 7I).

We continued to validate the clinical value of PLAUR in the GSE163154 cohort. GSVA results showed that the absolute enrichment of the hypoxia gene set in IPH was significantly increased compared to non-IPH (Fig. 7J). The boxplot showed that the expression of HIF1A was significantly increased in IPH compared to non-IPH (*p* = 0.031; Fig. 7K). Similarly, the characteristics of the GSE163154 dataset indicate that it is reliable as an external validation cohort. Subsequently, we detected the expression of PLAUR and found that the expression of PLAUR was significantly increased in IPH compared to non-IPH (*p* = 2.7e−05; Fig. 7L). The scatter plot showed that there was a significant positive correlation between HIF1A and PLAUR (r = 0.5941, *p* = 2.6e−05; Fig. 7M). The ROC analysis result showed that PLAUR can predict whether the samples in the GSE163154 cohort have plaque hemorrhage and its AUC value reaches 0.882 (Fig. 7N). These results extends the clinical value of PLAUR, suggesting that monitoring PLAUR levels in plaques may help predict the occurrence of vascular events.

### Construction and verification of the ceRNA network that regulates HIF1A.

**Figure 8:**
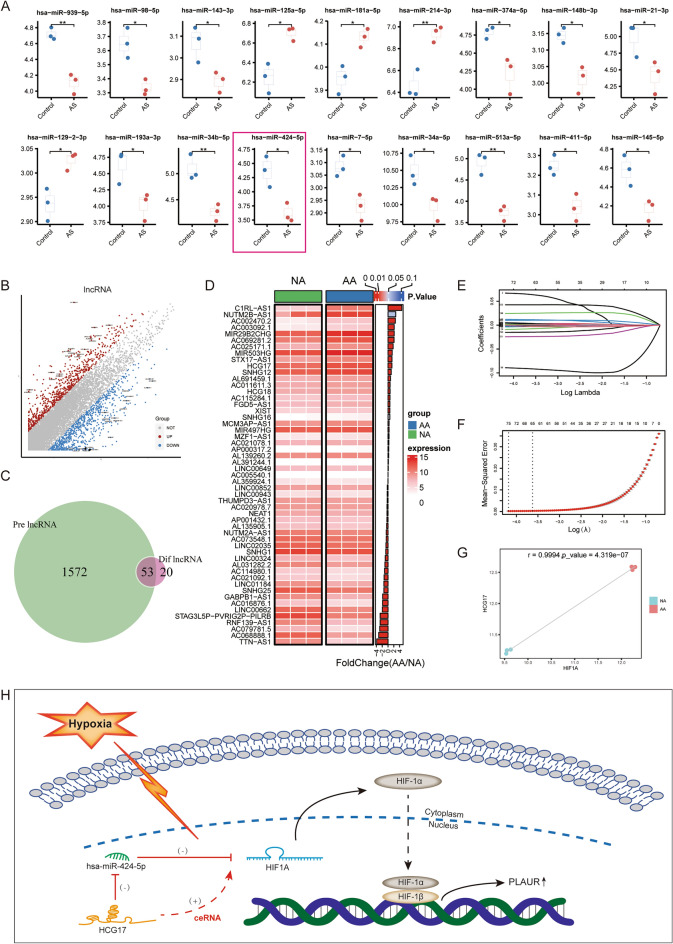
Construction and verification of the ceRNA network regulating HIF1A. (**A**) Boxplots showing all miRNAs with significant differential expression. (**B**) Volcano plot showing the differentially expressed lncRNAs between NA and AA in the GSE137580 cohort. |Log2FC|> 2 and *p* < 0.05 are considered to have a significant statistical difference. Up (red) and down (blue) are indicated. (**C**) Venn plot showing a total of 53 lncRNAs possessed probe information in the GSE97210 dataset. (**D**) Heatmap showing differential expression of lncRNA screened from the database between EA and AA. (**E**) LASSO coefficient profiles of the 53 lncRNAs in AS. (**F**) The log (lambda) sequence was used to construct a coefficient profile diagram. The LASSO model’s optimal parameter (lambda) was chosen. (**G**) Correlation between transcription level of HIF1A and expression of HCG17. (**H**) Schema summarizes that HCG17 and HSA-Mir-424-5p compete to regulate the expression level of HIF1A. PLAUR is the potential therapeutic target identified in this study, and HIF1A is its transcription factor.

The above experimental results support the conclusion that HIF1A may be a transcription factor of PLAUR. Considering that our study suggests that PLAUR is involved in the development of AS, we therefore sought to construct a possible ceRNA network regulating HIF1A, which will further influence the progression of lesions by regulating the expression of PLAUR thereby. We combined the prediction results from the TarBase dataset and miRTarBase dataset to identify 18 miRNAs supported by experimental evidence that may regulate HIF1A expression (S5A, Supplementary Table 4). We then validated the expression levels of these 18 miRNAs above in AS plaques on the dataset GSE13750. As shown in Fig. 8A, among these 18 predicted miRNAs, only hsa-miR-424-5p was significantly reduced in AS (as shown in the red box in Fig. 8A). Subsequently, to improve the confidence of the predictions, we used predictions from miRWalk and miRPath databases for cross-validation (Supplementary Tables 5, 6). The results showed that both datasets predicted HIF1A as a possible target gene for hsa-miR-424-5p (S5B). From the starBase database, we detected that 73 lncRNAs verified by at least three independent experiments can be combined with hsa-miR-424-5p (Supplementary Table 5). The GSE97210 cohort was selected as the validation set to test the predicted lncRNAs. With a threshold of *p* < 0.05 and |log2FC|> 2, 782 significantly upregulated lncRNAs and 863 significantly downregulated lncRNAs were found in AA samples from the GSE97210 dataset (Fig. 8B). Among the 73 predicted lncRNAs, only 53 possessed probe information in the GSE97210 dataset (Fig. 8C). Figure 8D showed the differential expression of these 53 lncRNAs between AA and NA. We further screened the lncRNAs most associated with plaque progression using LASSO regression. For LASSO algorithm, a more concise model was selected to construct the LASSO classifier after 10 cross-validation, and a total of 34 feature lncRNAs were identified (Fig. 8E,F). HCG17 is the only lncRNA to share the prediction results of the Starbase database and the LASSO analysis results. The scatter plot showed that there was a significant positive correlation between HIF1A and HCG17 (r = 0.9994, *p* = 4.3e−07; Fig. 8G). Through the screening and validation above, we constructed and mapped a possible ceRNA network that regulates HIF1A expression, and dysregulation of this network under pathological conditions may promote plaque development by upregulating PLAUR expression (Fig. 8H).

### Prediction and docking results of drugs targeting PLAUR

**Figure 9:**
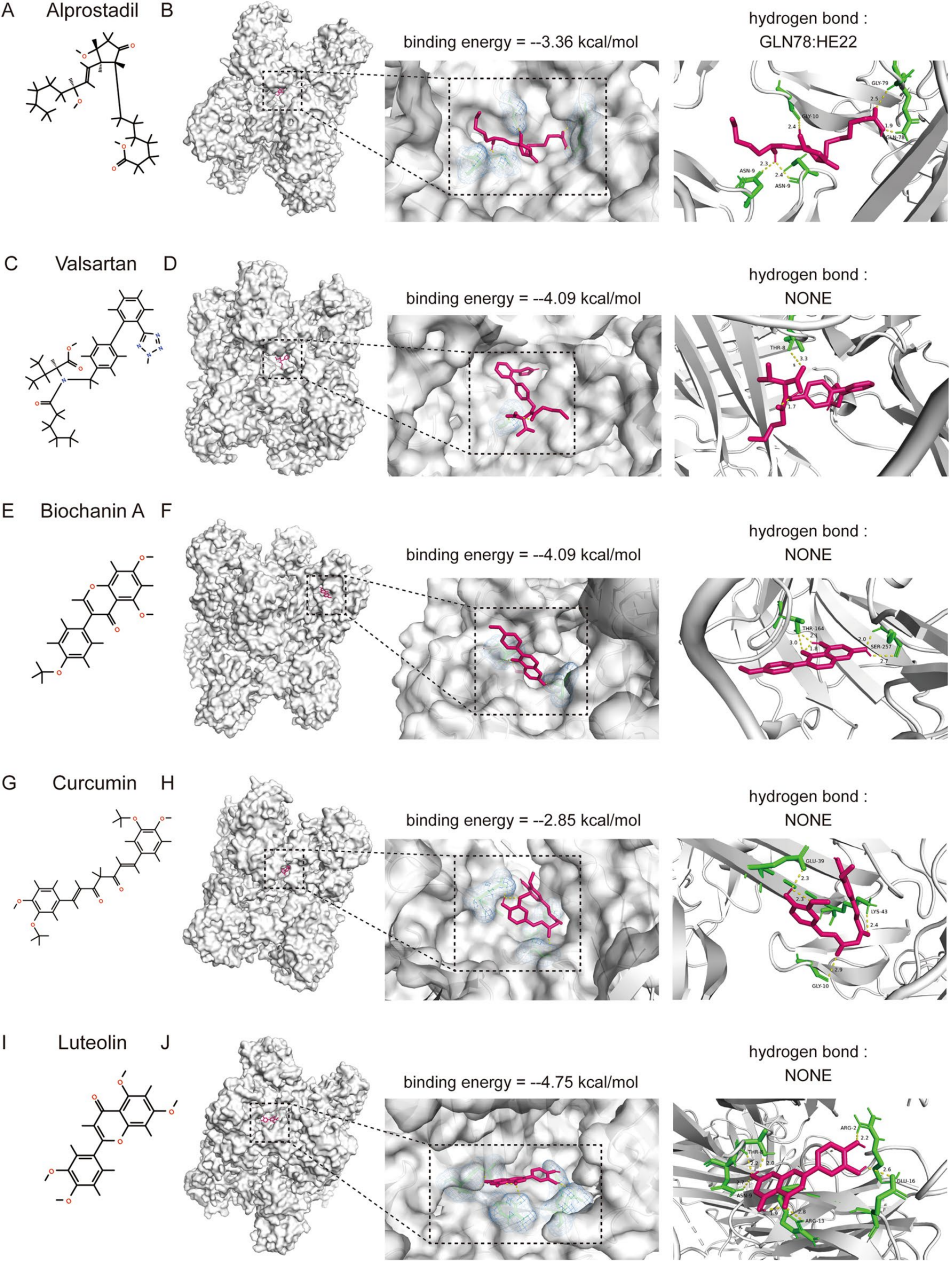
Prediction and docking results of the drugs targeting PLAUR. (**A**) Two-dimensional structure diagram of alprostadil. (**B**) Binding sites formed between alprostadil and PLAUR protein on ASN-9, GLY-10, GLY-79 and GLN-78, in which a hydrogen bond is formed on GLN-78. Pink represents alprostadil, while yellow represents the binding site of amino acid residues. (**C**) Two-dimensional structure diagram of valsartan. (**D**) Binding sites formed between valsartan and PLAUR protein on THR-8. Pink represents alprostadil, while yellow represents the binding site of amino acid residues. (**E**) Two-dimensional structure diagram of biochanin A. (**F**) Binding sites formed between biochanin A and PLAUR protein on THR-164 and SER-257. Pink represents biochanin A, while yellow represents the binding site of amino acid residues. (**G**) Two-dimensional structure diagram of curcumin. (**H**) Binding sites formed between curcumin and PLAUR protein on GLU-39, GLY-10 and LYS-43. Pink represents curcumin, while yellow represents the binding site of amino acid residues. (**I**) Two-dimensional structure diagram of luteolin. (**J**) Binding sites formed between luteolin and PLAUR protein on ARG-2, GLU-16, ARG-13, ASN-9 and THR-8. Pink represents luteolin, while yellow represents the binding site of amino acid residues.

We hope to screen for some drugs that can delay the AS development by blocking the biological function of PLAUR. First, using the DrugMatrix database, we found a total of 88 items describing the predicted small molecular compounds that could bind to PLAUR based on the standard of adjust *p* < 0.05 (Supplementary Table 6). Some of these small molecule compounds have been discussed in several items. To improve the clinical application value of our experiments, we focus our screening on commonly used drugs and natural products that have potential applications. Based on the above conditions, we finally recovered the following five drugs, including alprostadil, valsartan, biochanin A, luteolin, and curcumin. Figure 9A illustrates the two-dimensional structure of alprostadil. The results of auotodock show that the binding energy of alprostadil and PLAUR reaches − 3.36 kcal/mol. Molecular docking analysis predicted that alprostadil could interact with PLAUR proteins in ASN-9, GLY-10, GLY-79, and GLN-78. Meanwhile, one hydrogen bond is formed on GLN-78 (Fig. 9B). Figure 9C illustrates the two-dimensional structure of valsartan. The results of auotodock show that the binding energy of valsartan and PLAUR reaches − 4.09 kcal/mol. Molecular docking analysis predicted that alprostadil could interact with PLAUR proteins in THR-8 and no hydrogen bond was formed (Fig. 9D). Figure 9E illustrates the two-dimensional structure of biochanin A. The results of auotodock show that the binding energy of biochanin A and PLAUR reaches − 4.19 kcal/mol. Molecular docking analysis predicted that biochanin A could interact with PLAUR proteins in THR-164 and SER-257 and no hydrogen bond formed (Fig. 9F). Figure 9G illustrates the two-dimensional structure of curcumin. The results of auotodock show that the binding energy of curcumin and PLAUR reaches − 2.85 kcal/mol. Molecular docking analysis predicted that curcumin could interact with the PLAUR proteins in GLU-39, GLY-10, and LYS-43 and no hydrogen bond formed (Fig. 9H). Figure 9I illustrates the two-dimensional structure of luteolin. The results of auotodock show that the binding energy of luteolin and PLAUR reaches − 4.75 kcal/mol. Molecular docking predicted that luteolin could interact with PLAUR protein in ARG-2, GLU-16, ARG-13, ASN-9 and THR-8, but no hydrogen bond was formed (Fig. 9J). In conclusion, all five selected drugs have an excellent binding capacity to PALUR. This indicates that these drugs may reduce the hypoxia stress of AA by inhibiting PLAUR function.

## Discussion

Numerous biological processes, such as metabolism, angiogenesis, and tumor metastasis, are significantly impacted by hypoxia. Hypoxia causes a complicated array of chemical reactions at the cellular level, mainly dependent on the central function of the transcription factor HIF1A^32^. In AS plaques, evidence has been found for interactions between hypoxia and other pathways that promote the progression of lesions^7^. This suggests that hypoxia may lead to poor therapeutic efficacy and adverse clinical outcomes. Hypoxia seems to make a lot of sense as an emerging biomarker and treatment target for AS based on these observations. The progression of AS frequently coincides with changes in the expression of a number of genes, among which genes related to hypoxia have sparked a great deal of attention. Given the quickening development of sequencing technology, high-throughput genomics has improved the ability of researchers to find genes that promote the progression of AS^33^.

In this study, GSEA analysis results based on transcriptome data demonstrated the enrichment of hypoxia in AA plaques. And scRNA-seq data analysis showed that hypoxia was significantly enriched in macrophage clusters compared to other cell clusters, which is consistent with previous evidence of hypoxia observed in symptomatic patients undergoing carotid endarterectomy^5^. The WGCNA results identified a module with a significant positive correlation with the phenotype of AA plaques, characterized by HIF1A located in the central region of the module. A significant enrichment of the hypoxia response was revealed by GO analysis of the genes in the module. We call the genes contained in this module hypoxia-related genes. Our research provides evidences from the perspective of bioinformatics to prove that hypoxia is one of the features of AS lesions.

The continuously activated and uncontrolled inflammatory state in AS plaque is mediated in part by resident long-term resident immune cells. Chronic low-grade inflammation may lead to a high metabolic state of cells, which, together with the increased oxygen consumption of immune cells, leads to an imbalance in the oxygen supply of inflammatory tissues. Our analysis of immunocyte infiltration showed that there was extensive and significant immunocyte infiltration in AA. This may indicate that the fiber cap reduces the diffusion of oxygen and the enrichment of immune cells co-mediates the environment of hypoxia in AA. HIF1A has been showed to regulate the migration of some cells in tumor tissues. Based on the above facts, we hope to clarify the interaction between hypoxic microenvironment and immune infiltration in plaque. Our results suggest that HIF1A may mediate increased macrophage infiltration and negatively regulate beneficial Treg cell infiltration. This may be the molecular basis of hypoxia that promotes plaque progression. For the first time, we demonstrated the mechanism of interaction between the hypoxia microenvironment and immune cell infiltration in the AS plaque at the global and molecular levels through bioinformatic methods.

The innate immune response mediated by monocyte-macrophages induced by lipoproteins triggers uncontrolled inflammation of AS, and then activates the adaptive immune system composed of B cells and T cells to maintain long-term inflammation activation^34^. Our results show that the heterogeneity of immune cell infiltration between EA and AA is reflected primarily in macrophages and lymphocytes. Consistent with previous reports^35^, we found that macrophages were the main infiltrating immune cells in AS plaques and that the abundance of macrophages in AA is significantly higher than in EA. Although the number of T cells, B cells and NK cells is less than that of macrophages, they show strong heterogeneity between EA and AA. Our correlation analysis suggests that HIF1A may help macrophages infiltrate the plaque. Our scRNA-seq data analysis showed that HIF1A was highly expressed locally in macrophage clusters, supporting the findings of the correlation analysis. The basis of macrophages initiating tissue migration in response to hypoxia is through HIF-1α Induces pyruvate dehydrogenase kinase isoenzyme 1^36^. We note that HIF1A negatively regulates Treg cell infiltration, but several studies revealed a protective role for Treg cells against AS^37^. In the progression of AS, the interaction of hypoxia and immune cell infiltration contributes to the inflammatory phenotype and therapeutic resistance, which may lead to plaque progression and poor clinical outcomes^38^. The results of our analysis showed that plaques with higher hypoxia environments have a higher ImmuneScore. The implications of these observations seem to explain why hypoxia is emerging as a potential biomarker and target in the treatment of AS^39^.

Machine learning based algorithms are increasingly used in clinical decision making, and the random forest algorithm is often used to predict key dependent variables in different phenotypes of the same disease^40^. In this study, PLAUR is finally determined as the target gene by integrating the results of the random forest algorithm and the WGCNA analysis. The use of public databases to predict potential target genes of transcription factors has been adopted by numerous studies. In this study, we used cross-validation of multiple databases in order to enhance the stability of prediction in a high-throughput manner. The predicted results suggest that HIF1A may be a transcription factor of PLAUR. There is necessarily a parallel relationship between transcription factors and target genes at the expression level as well as a co-localization feature at the cellular level. We validated the correlation between HIF1A and PLAUR expression levels in AS lesions on multiple sequencing datasets. In particular, we confirmed on single-cell data that HIF1A and PLAUR have a stable co-localization feature on macrophage clusters. Our experimental results provide non-direct evidence to support our predictions. Nevertheless, in vivo and in vitro experiments are still necessary to determine transcriptional relationships and need to be supplemented with future experiments. Similar to AS, hypoxia is also common in the subcutaneous white adipose tissue of obese patients and mediates macrophage residency and phenotypic changes^41^. Previous studies found that after weight loss surgery, the PLAUR expression was negatively regulated and macrophage infiltration was reduced, indicating that PLAUR may participate in macrophage attraction and regulation in the adipose tissue of morbidly obese patients^42^. The microenvironment of hypoxia is also common in various tumors. Hypoxia is associated with aggressive growth and metastasis of tumors, which may be an unfavorable prognostic factor, and is associated with a shorter survival period in some tumors^43^. Hypoxia increases the tumor cells invasion by upregulating PLAUR expression^44,45^. These results suggest that PLAUR may be a potential means of treating AS by inhibiting immune and inflammatory responses, which deserves further experimental verification. However, studies of PLAUR in AS are still very limited. Consistent with these studies, we also observed synergistic high expression of PLAUR and HIF1A. ROC analysis showed that PLAUR could well distinguish AA from EA, indicating its potential diagnostic ability. IPH is one of the outcomes of AA. There is a theory that IPH results from new immature blood vessels that respond specifically to hypoxia stimuli when they are formed^46^. ROC analysis shows that PLAUR can well distinguish IPH from non-IPH, which broadens the possibility of its clinical application. Our findings highlight the potential of PLAUR as a novel biomarker and therapeutic target for AS.

Our study suggested that HIF1A may be a transcription factor for PLAUR, so we further explored the possible ceRNA network regulating HIF1A. Evidence is gathering that dysregulation of miRNA expression is related to various pathological processes in AS. We used cross-prediction results from multiple public databases storing CHIP-seq data and performed expression validation using the dataset, and finally identified hsa-miR-424-5p as a possible miRNA regulating HIF1A. Some studies have suggested that Mir-424-5p plays an instrumental role in the development of CVD. An in vitro study of miR-424 showed that overexpression of miR-424 inhibited the proliferation of pulmonary artery endothelial cells^47^. In addition, the upregulation of plasma miR-424-5p is associated with a higher incidence of vascular events, such as deep vein thrombosis and venous thromboembolism^48,49^. MiR-424-5p may be a biomarker of the onset of subclinical CVD^50^. Furthermore, some bioinformatic-based studies have shown that mir-424-5p seems to be a marker of plaque instability^51,52^. Our analysis shows that in vivo and in vitro studies are urgently needed to find evidence of direct binding of Hsa-miR-424-5p and HIF1A to AS-related cells. More and more evidence shows that the lncRNA-miRNA-mRNA axis, also known as the ceRNA network, plays a critical role in the development of several forms of CVD^53^. In this study, we used the starBase database to predict lncRNA that can bind to hsa-miR-424-5p. Using LASSO, we screened lncRNAs closely related to AA in the GSE97210 dataset. The overlap result was HCG17. HCG17 has been identified as a very effective biomarker of ischemic stroke^54^. Our study provides direction to uncover the ceRNA network that regulates plaque progression. Although bioinformatics studies can help predict potential regulatory networks, reliable in vivo and in vitro experiments are the only direct evidence to confirm the relationship between these key nodes on the ceRNA regulatory network. Future robust experimental results are needed to support our conclusions.

Through the prediction of the DSigDB database and the screening of AutoDock, we finally selected five drugs targeting PLAUR, namely alprostadil, valsartan, biochanin A, curcumin and luteolin. Alprostadil promoted the stability of plaques in the rabbit model in a dose-dependent manner^55^. Valsartan treatment has been reported to inhibit plaques formation by inhibiting the expression of proinflammatory genes and reducing plaque lipid content^56^. Some studies have shown that biochanin A has a protective effect on AS by decreasing blood lipid levels^57^, reducing lipid accumulation and the inflammatory response^58^. Curcumin has a hypolipidemic effect and a significant antioxidant capacity, which can prevent the development of plaques by reducing lipid peroxidation and oxLDL production^59^. Luteolin treatment can inhibit foam cell formation and macrophage apoptosis by promoting autophagy, which may have the potential to be used in the treatment of AS^60^. Our study revealed that these drugs may alleviate hypoxic stress by inhibiting PLAUR, thereby improving plaques. This provides a basis for the search for new indications for drugs marketed such as desartan and alprostadil. At the same time, there is already partial evidence to support that extracts from natural plants such as luteolin, curcumin, and biochanin A may be candidates for the treatment of AS. Our findings add new evidence that will aid in the development of these drugs.

Our study also suffers from the limitations of bioinformatics research. First, despite using as many datasets as possible for thorough validation, patch heterogeneity and sampling bias brought on by cross-platform studies are still present. In addition, PLAUR as a potential diagnostic and therapeutic target needs to be confirmed by further in vivo and in vitro experiments. Both the ceRNA network we constructed and the mechanism of how the five drugs we screened act on PLAUR and slow the development of plaques require evidence from basic research.

## Conclusions

This resaerch combined bioinformatics and machine learning methods to reveal the hypoxia characteristics of AS, and screened PLAUR, a biomarker related to AS. We constructed a possible ceRNA network HCG17-miR-424-5p-HIF1A involved in plaque formation and development by regulating the transcription of PLAUR. Drug database screening and autodock validation found that prostaglandin, valsartan, biotin A, luteolin and curcumin may be potential targeted drugs for PLAUR. The PLAUR identified in this study can provide a potential target for the diagnosis and treatment for AS. As a reliable biomarker and therapeutic target of AS, the extent to which upregulation of PLAUR promotes the development of AS remains to be studied.

## Supplementary Information

Supplementary Information.

## Data Availability

All data generated or analysed during this study are included in this published article (and its Supplementary Information files). Publicly available datasets analysed during the current study are available in the GEO database under accession codes: GSE28829 (https://www.ncbi.nlm.nih.gov/geo/query/acc.cgi?acc=GSE28829);GSE43292 (https://www.ncbi.nlm.nih.gov/geo/query/acc.cgi?acc=GSE43292);GSE163154 (https://www.ncbi.nlm.nih.gov/geo/query/acc.cgi?acc=GSE163154); GSE97210 (https://www.ncbi.nlm.nih.gov/geo/query/acc.cgi?acc=GSE97210); GSE137580 (https://www.ncbi.nlm.nih.gov/geo/query/acc.cgi?acc=GSE137580); GSE155512 (https://www.ncbi.nlm.nih.gov/geo/query/acc.cgi?acc=GSE155512); GSE159677 (https://www.ncbi.nlm.nih.gov/geo/query/acc.cgi?acc=GSE159677); GSE131778 (https://www.ncbi.nlm.nih.gov/geo/query/acc.cgi?acc=GSE131778); GSE137581 (https://www.ncbi.nlm.nih.gov/geo/query/acc.cgi?acc=GSE137581); GSE69187 (https://www.ncbi.nlm.nih.gov/geo/query/acc.cgi?acc=GSE69187); GSE72248 (https://www.ncbi.nlm.nih.gov/geo/query/acc.cgi?acc=GSE72248); GSE76812 (https://www.ncbi.nlm.nih.gov/geo/query/acc.cgi?acc=GSE76812); GSE10000 (https://www.ncbi.nlm.nih.gov/geo/query/acc.cgi?acc=GSE10000).

## Abbreviations

AS: Atherosclerosis
AA: Advanced-stage plaques
AUC: Area under the curve
ceRNA: Competing endogenous RNA
CI: Confidence intervals
CVD: Cardiovascular disease
DEG: Differentially expressed gene
EA: Early-stage plaques
EC: Endothelial cell
FC: Fibroblast cell
GSEA: Gene set enrichment analysis
GO: Gene ontology
GSVA: Gene set variation analysis
HIF1A: Hypoxia inducible factor 1 subunit alpha
IPH: Intraplaque haemorrhage
ICS: Intermediate cell state
KEGG: Kyoto encyclopedia genes and genomes
LASSO: Least absolute shrinkage and selection operator
NA: Normal intima tissues
PCA: Principal component analysis
PLAUR: Plasminogen activator, urokinase receptor
ROC: Receiver operating characteristic
SMC: Smooth muscle cell
ssGSEA: Single sample gene set enrichment analysis
sc-RNA seq: Single-cell RNA sequencing
UMPA: Uniform manifold approximation and projection
WGCNA: Weighted gene co-expression network analysis

## References

[1] Basatemur GL, Jørgensen HF, Clarke MCH, Bennett MR, Mallat Z (2019). Vascular smooth muscle cells in atherosclerosis. Nat. Rev. Cardiol..

[2] Borén J, Chapman MJ, Krauss RM, Packard CJ, Bentzon JF, Binder CJ, Daemen MJ, Demer LL, Hegele RA, Nicholls SJ (2020). Low-density lipoproteins cause atherosclerotic cardiovascular disease: Pathophysiological, genetic, and therapeutic insights: A consensus statement from the European atherosclerosis society consensus panel. Eur. Heart J..

[3] Baigent C, Blackwell L, Emberson J, Holland LE, Reith C, Bhala N, Peto R, Barnes EH, Keech A, Simes J (2010). Efficacy and safety of more intensive lowering of LDL cholesterol: A meta-analysis of data from 170,000 participants in 26 randomised trials. Lancet.

[4] Kwan AC, Aronis KN, Sandfort V, Blumenthal RS, Bluemke DA (2017). Bridging the gap for lipid lowering therapy: Plaque regression, coronary computed tomographic angiography, and imaging-guided personalized medicine. Expert Rev. Cardiovasc. Ther..

[5] Sluimer JC, Gasc JM, van Wanroij JL, Kisters N, Groeneweg M, Sollewijn Gelpke MD, Cleutjens JP, van den Akker LH, Corvol P, Wouters BG (2008). Hypoxia, hypoxia-inducible transcription factor, and macrophages in human atherosclerotic plaques are correlated with intraplaque angiogenesis. J. Am. Coll. Cardiol..

[6] Björnheden T, Levin M, Evaldsson M, Wiklund O (1999). Evidence of hypoxic areas within the arterial wall in vivo. Arterioscler. Thromb. Vasc. Biol..

[7] Hultén LM, Levin M (2009). The role of hypoxia in atherosclerosis. Curr. Opin. Lipidol..

[8] Vink A, Schoneveld AH, Lamers D, Houben AJ, van der Groep P, van Diest PJ, Pasterkamp G (2007). HIF-1 alpha expression is associated with an atheromatous inflammatory plaque phenotype and upregulated in activated macrophages. Atherosclerosis.

[9] Knutson AK, Williams AL, Boisvert WA, Shohet RV (2021). HIF in the heart: development, metabolism, ischemia, and atherosclerosis. J. Clin. Invest..

[10] Ye Z, Wang XK, Lv YH, Wang X, Cui YC (2022). The integrated analysis identifies three critical genes as novel diagnostic biomarkers involved in immune infiltration in atherosclerosis. Front. Immunol..

[11] Saenz-Pipaon G, Ravassa S, Larsen KL, Martinez-Aguilar E, Orbe J, Rodriguez JA, Fernandez-Alonso L, Gonzalez A, Martín-Ventura JL, Paramo JA (2022). Lipocalin-2 and calprotectin potential prognosis biomarkers in peripheral arterial disease. Eur. J. Vasc. Endovasc. Surg..

[12] Döring Y, Manthey HD, Drechsler M, Lievens D, Megens RT, Soehnlein O, Busch M, Manca M, Koenen RR, Pelisek J (2012). Auto-antigenic protein-DNA complexes stimulate plasmacytoid dendritic cells to promote atherosclerosis. Circulation.

[13] Ayari H, Bricca G (2013). Identification of two genes potentially associated in iron-heme homeostasis in human carotid plaque using microarray analysis. J. Biosci..

[14] Jin H, Goossens P, Juhasz P, Eijgelaar W, Manca M, Karel JMH, Smirnov E, Sikkink C, Mees BME, Waring O (2021). Integrative multiomics analysis of human atherosclerosis reveals a serum response factor-driven network associated with intraplaque hemorrhage. Clin. Transl. Med..

[15] Hu YW, Guo FX, Xu YJ, Li P, Lu ZF, McVey DG, Zheng L, Wang Q, Ye JH, Kang CM (2019). Long noncoding RNA NEXN-AS1 mitigates atherosclerosis by regulating the actin-binding protein NEXN. J. Clin. Invest..

[16] Pan H, Xue C, Auerbach BJ, Fan J, Bashore AC, Cui J, Yang DY, Trignano SB, Liu W, Shi J (2020). Single-cell genomics reveals a novel cell state during smooth muscle cell phenotypic switching and potential therapeutic targets for atherosclerosis in mouse and human. Circulation.

[17] Alsaigh T, Evans D, Frankel D, Torkamani A (2022). Decoding the transcriptome of calcified atherosclerotic plaque at single-cell resolution. Commun. Biol..

[18] Wirka RC, Wagh D, Paik DT, Pjanic M, Nguyen T, Miller CL, Kundu R, Nagao M, Coller J, Koyano TK (2019). Atheroprotective roles of smooth muscle cell phenotypic modulation and the TCF21 disease gene as revealed by single-cell analysis. Nat. Med..

[19] Du W, Wong C, Song Y, Shen H, Mori D, Rotllan N, Price N, Dobrian AD, Meng H, Kleinstein SH (2016). Age-associated vascular inflammation promotes monocytosis during atherogenesis. Aging Cell.

[20] Li YF, Huang X, Li X, Gong R, Yin Y, Nelson J, Gao E, Zhang H, Hoffman NE, Houser SR (2016). Caspase-1 mediates hyperlipidemia-weakened progenitor cell vessel repair. Front. Biosci. (Landmark Ed).

[21] Moreno-Viedma V, Amor M, Sarabi A, Bilban M, Staffler G, Zeyda M, Stulnig TM (2016). Common dysregulated pathways in obese adipose tissue and atherosclerosis. Cardiovasc. Diabetol..

[22] Gräbner R, Lötzer K, Döpping S, Hildner M, Radke D, Beer M, Spanbroek R, Lippert B, Reardon CA, Getz GS (2009). Lymphotoxin beta receptor signaling promotes tertiary lymphoid organogenesis in the aorta adventitia of aged ApoE−/− mice. J. Exp. Med..

[23] Ritchie ME, Phipson B, Wu D, Hu Y, Law CW, Shi W, Smyth GK (2015). limma powers differential expression analyses for RNA-sequencing and microarray studies. Nucl. Acids Res..

[24] Wilkerson MD, Hayes DN (2010). ConsensusClusterPlus: A class discovery tool with confidence assessments and item tracking. Bioinformatics.

[25] Subramanian A, Tamayo P, Mootha VK, Mukherjee S, Ebert BL, Gillette MA, Paulovich A, Pomeroy SL, Golub TR, Lander ES (2005). Gene set enrichment analysis: A knowledge-based approach for interpreting genome-wide expression profiles. Proc. Natl. Acad. Sci. U. S. A..

[26] Langfelder P, Horvath S (2008). WGCNA: An R package for weighted correlation network analysis. BMC Bioinform..

[27] Hänzelmann S, Castelo R, Guinney J (2013). GSVA: gene set variation analysis for microarray and RNA-seq data. BMC Bioinform..

[28] Charoentong P, Finotello F, Angelova M, Mayer C, Efremova M, Rieder D, Hackl H, Trajanoski Z (2017). Pan-cancer immunogenomic analyses reveal genotype-immunophenotype relationships and predictors of response to checkpoint blockade. Cell Rep..

[29] Aibar S, González-Blas CB, Moerman T, Huynh-Thu VA, Imrichova H, Hulselmans G, Rambow F, Marine JC, Geurts P, Aerts J (2017). SCENIC: Single-cell regulatory network inference and clustering. Nat. Methods.

[30] Engebretsen S, Bohlin J (2019). Statistical predictions with glmnet. Clin. Epigenetics.

[31] Forli S, Huey R, Pique ME, Sanner MF, Goodsell DS, Olson AJ (2016). Computational protein-ligand docking and virtual drug screening with the AutoDock suite. Nat. Protoc..

[32] Majmundar AJ, Wong WJ, Simon MC (2010). Hypoxia-inducible factors and the response to hypoxic stress. Mol. Cell.

[33] Wu X, Qin K, Iroegbu CD, Xiang K, Peng J, Guo J, Yang J, Fan C (2022). Genetic analysis of potential biomarkers and therapeutic targets in ferroptosis from coronary artery disease. J. Cell Mol. Med..

[34] Tabas I, Lichtman AH (2017). Monocyte-macrophages and T cells in atherosclerosis. Immunity.

[35] Moore KJ, Sheedy FJ, Fisher EA (2013). Macrophages in atherosclerosis: a dynamic balance. Nat. Rev. Immunol..

[36] Semba H, Takeda N, Isagawa T, Sugiura Y, Honda K, Wake M, Miyazawa H, Yamaguchi Y, Miura M, Jenkins DM (2016). HIF-1α-PDK1 axis-induced active glycolysis plays an essential role in macrophage migratory capacity. Nat. Commun..

[37] Ait-Oufella H, Salomon BL, Potteaux S, Robertson AK, Gourdy P, Zoll J, Merval R, Esposito B, Cohen JL, Fisson S (2006). Natural regulatory T cells control the development of atherosclerosis in mice. Nat. Med..

[38] Yu B, Wang X, Song Y, Xie G, Jiao S, Shi L, Cao X, Han X, Qu A (2022). The role of hypoxia-inducible factors in cardiovascular diseases. Pharmacol. Ther..

[39] Jain T, Nikolopoulou EA, Xu Q, Qu A (2018). Hypoxia inducible factor as a therapeutic target for atherosclerosis. Pharmacol. Ther..

[40] Shen B, Yi X, Sun Y, Bi X, Du J, Zhang C, Quan S, Zhang F, Sun R, Qian L (2020). Proteomic and metabolomic characterization of COVID-19 patient sera. Cell.

[41] Kabon B, Nagele A, Reddy D, Eagon C, Fleshman JW, Sessler DI, Kurz A (2004). Obesity decreases perioperative tissue oxygenation. Anesthesiology.

[42] Cancello R, Henegar C, Viguerie N, Taleb S, Poitou C, Rouault C, Coupaye M, Pelloux V, Hugol D, Bouillot JL (2005). Reduction of macrophage infiltration and chemoattractant gene expression changes in white adipose tissue of morbidly obese subjects after surgery-induced weight loss. Diabetes.

[43] Lin W, Wu S, Chen X, Ye Y, Weng Y, Pan Y, Chen Z, Chen L, Qiu X, Qiu S (2020). Characterization of hypoxia signature to evaluate the tumor immune microenvironment and predict prognosis in glioma groups. Front. Oncol..

[44] Rofstad EK, Mathiesen B, Galappathi K (2004). Increased metastatic dissemination in human melanoma xenografts after subcurative radiation treatment: Radiation-induced increase in fraction of hypoxic cells and hypoxia-induced up-regulation of urokinase-type plasminogen activator receptor. Cancer Res..

[45] Lee KH, Choi EY, Hyun MS, Kim JR (2004). Involvement of MAPK pathway in hypoxia-induced up-regulation of urokinase plasminogen activator receptor in a human prostatic cancer cell line, PC3MLN4. Exp. Mol. Med..

[46] Michel JB, Martin-Ventura JL, Nicoletti A, Ho-Tin-Noé B (2014). Pathology of human plaque vulnerability: Mechanisms and consequences of intraplaque haemorrhages. Atherosclerosis.

[47] Kim J, Kang Y, Kojima Y, Lighthouse JK, Hu X, Aldred MA, McLean DL, Park H, Comhair SA, Greif DM (2013). An endothelial apelin-FGF link mediated by miR-424 and miR-503 is disrupted in pulmonary arterial hypertension. Nat. Med..

[48] Starikova I, Jamaly S, Sorrentino A, Blondal T, Latysheva N, Sovershaev M, Hansen JB (2015). Differential expression of plasma miRNAs in patients with unprovoked venous thromboembolism and healthy control individuals. Thromb. Res..

[49] Wang X, Sundquist K, Elf JL, Strandberg K, Svensson PJ, Hedelius A, Palmer K, Memon AA, Sundquist J, Zöller B (2016). Diagnostic potential of plasma microRNA signatures in patients with deep-vein thrombosis. Thromb. Haemost..

[50] Tamara A, Coulson DJ, Latief JS, Bakhashab S, Weaver JU (2021). Upregulated anti-angiogenic miR-424-5p in type 1 diabetes (model of subclinical cardiovascular disease) correlates with endothelial progenitor cells, CXCR1/2 and other parameters of vascular health. Stem Cell Res. Ther..

[51] Andreou I, Sun X, Stone PH, Edelman ER, Feinberg MW (2015). miRNAs in atherosclerotic plaque initiation, progression, and rupture. Trends Mol. Med..

[52] Aquila G, Fortini C, Pannuti A, Delbue S, Pannella M, Morelli MB, Caliceti C, Castriota F, de Mattei M, Ongaro A (2017). Distinct gene expression profiles associated with Notch ligands Delta-like 4 and Jagged1 in plaque material from peripheral artery disease patients: A pilot study. J. Transl. Med..

[53] Huang Y (2018). The novel regulatory role of lncRNA-miRNA-mRNA axis in cardiovascular diseases. J. Cell Mol. Med..

[54] Zheng Y, Sun S, Yu M, Fu X (2019). Identification of potential hub-lncRNAs in ischemic stroke based on Subpathway-LNCE method. J. Cell Biochem..

[55] Bai W, Zheng X, Zhou L, Li H (2012). Prostaglandin E1 dose-dependently promotes stability of atherosclerotic plaque in a rabbit model. Can. J. Physiol. Pharmacol..

[56] Zhang H, Liu G, Zhou W, Zhang W, Wang K, Zhang J (2019). Neprilysin inhibitor-angiotensin II receptor blocker combination therapy (Sacubitril/valsartan) suppresses atherosclerotic plaque formation and inhibits inflammation in apolipoprotein E- deficient mice. Sci. Rep..

[57] Howes JB, Sullivan D, Lai N, Nestel P, Pomeroy S, West L, Eden JA, Howes LG (2000). The effects of dietary supplementation with isoflavones from red clover on the lipoprotein profiles of post menopausal women with mild to moderate hypercholesterolaemia. Atherosclerosis.

[58] Yu XH, Chen JJ, Deng WY, Xu XD, Liu QX, Shi MW, Ren K (2020). Biochanin a mitigates atherosclerosis by inhibiting lipid accumulation and inflammatory response. Oxid. Med. Cell Longev..

[59] Li H, Sureda A, Devkota HP, Pittalà V, Barreca D, Silva AS, Tewari D, Xu S, Nabavi SM (2020). Curcumin, the golden spice in treating cardiovascular diseases. Biotechnol. Adv..

[60] Zhang BC, Zhang CW, Wang C, Pan DF, Xu TD, Li DY (2016). Luteolin attenuates foam cell formation and apoptosis in Ox-LDL-stimulated macrophages by enhancing autophagy. Cell Physiol. Biochem..

