# Supplementary material for: Comprehensive analysis of the diagnostic and therapeutic value of the hypoxia-related gene PLAUR in the progression of atherosclerosis
Source: Sci Rep. 2023 May 26;13:8533. doi: 10.1038/s41598-023-35548-z (PMC10220023; doi:10.1038/s41598-023-35548-z)
Supplement: Supplementary file 1 — Supplementary Information. [file 41598_2023_35548_MOESM1_ESM.pdf]

Comprehensive analysis of the diagnostic and therapeutic value of hypoxia-related gene PLAUR in atherosclerosis progression

Chengyi Dai[1, †,*], Yuhang Lin[2, †]

[1] *The First People's Hospital of Xiaoshan District, Hangzhou, Zhejiang 311200, China;*

[2]*Department of Neurology, Wenling First People's Hospital, The Affiliated Wenling Hospital of Wenzhou Medical University, Wenling, 317500 Zhejiang Province, China*

Corresponding authors

[†]These authors have contributed equally to this work and share first authorship.

Correspondence should be addressed to Chengyi Dai*; dcy01281996@163.com, at the *The First People's Hospital of Xiaoshan District, Hangzhou, Zhejiang 311200, China.*

Supplementary Materials

- **Supplementary Figures**

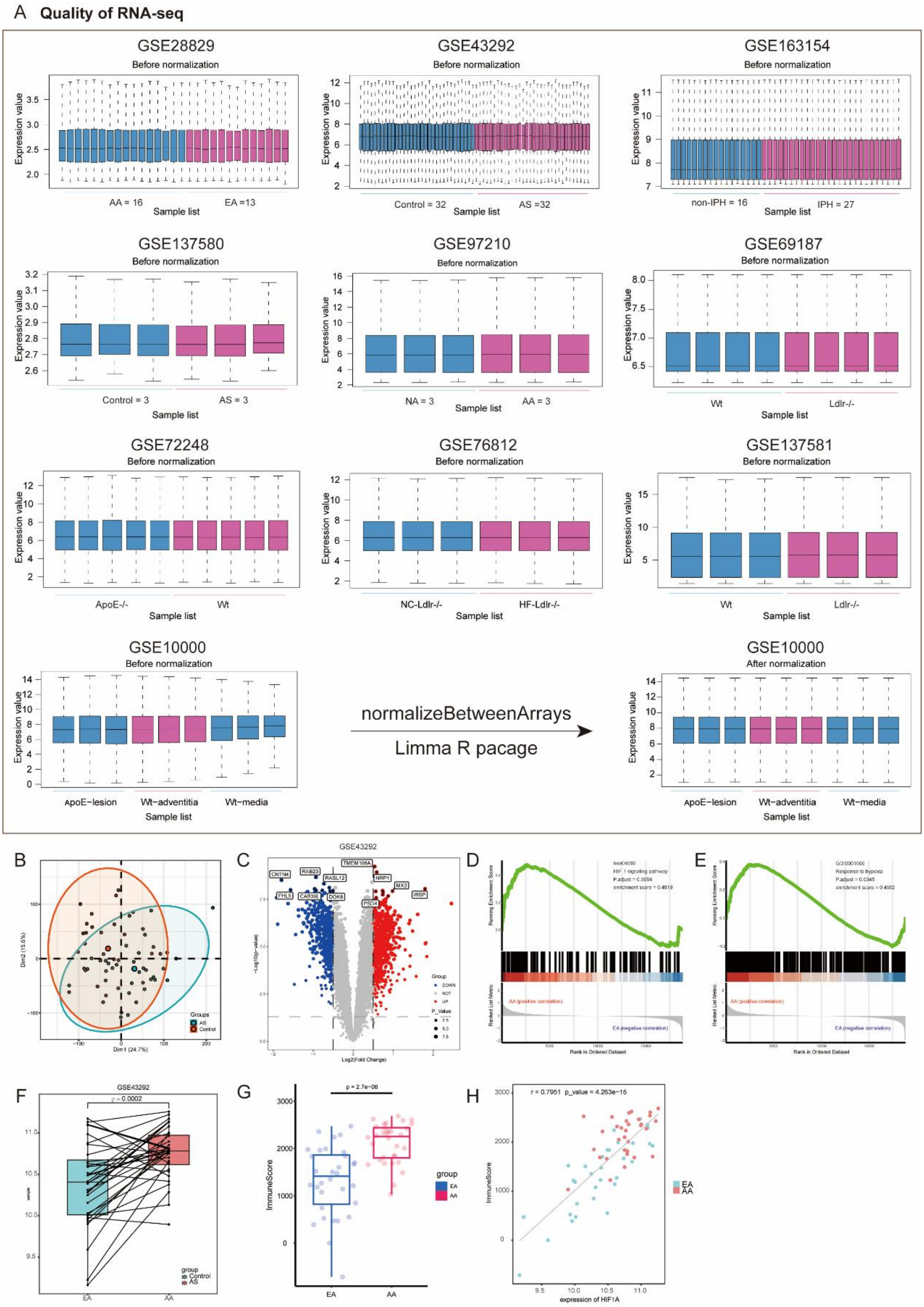

Supplementary Figure S1. Validation of the applicability of the validation cohort. (A) Quality control of incorporated RNA-seq cohorts. (B) EA and AA were clustered into two clusters with significant heterogeneity in the results of PCA analysis. (C) Volcano plot showing the DEGs between EA and AA in the GSE43292 cohort. |Log2FC| > 0.5 and p < 0.05 are considered to have a

significant statistical difference. Up (red) and down (blue) are indicated. (D) GSEA of 105 genes represents the HIF-1 signal pathway, which reveals the relationship between plaque status and HIF-1 pathway. AA is located on the left side near the starting point of the x-axis, while EA is located on the right side of the x-axis (GSE43292). (E) GSEA of 289 marker genes represents "hypoxia response", indicating the relationship between plaque status and hypoxia response. AA is located on the left side near the origin of the x-axis, while EA is located on the right side of the x-axis (GSE43292). (F) The transcription level of HIF1A was significantly increased in AA ($p = 0.0002$). (G) ImmuneScore was significantly increased in AA ($p = 2.7e-6$). (H) Correlation between HIF1A transcription levels and ImmuneScore.

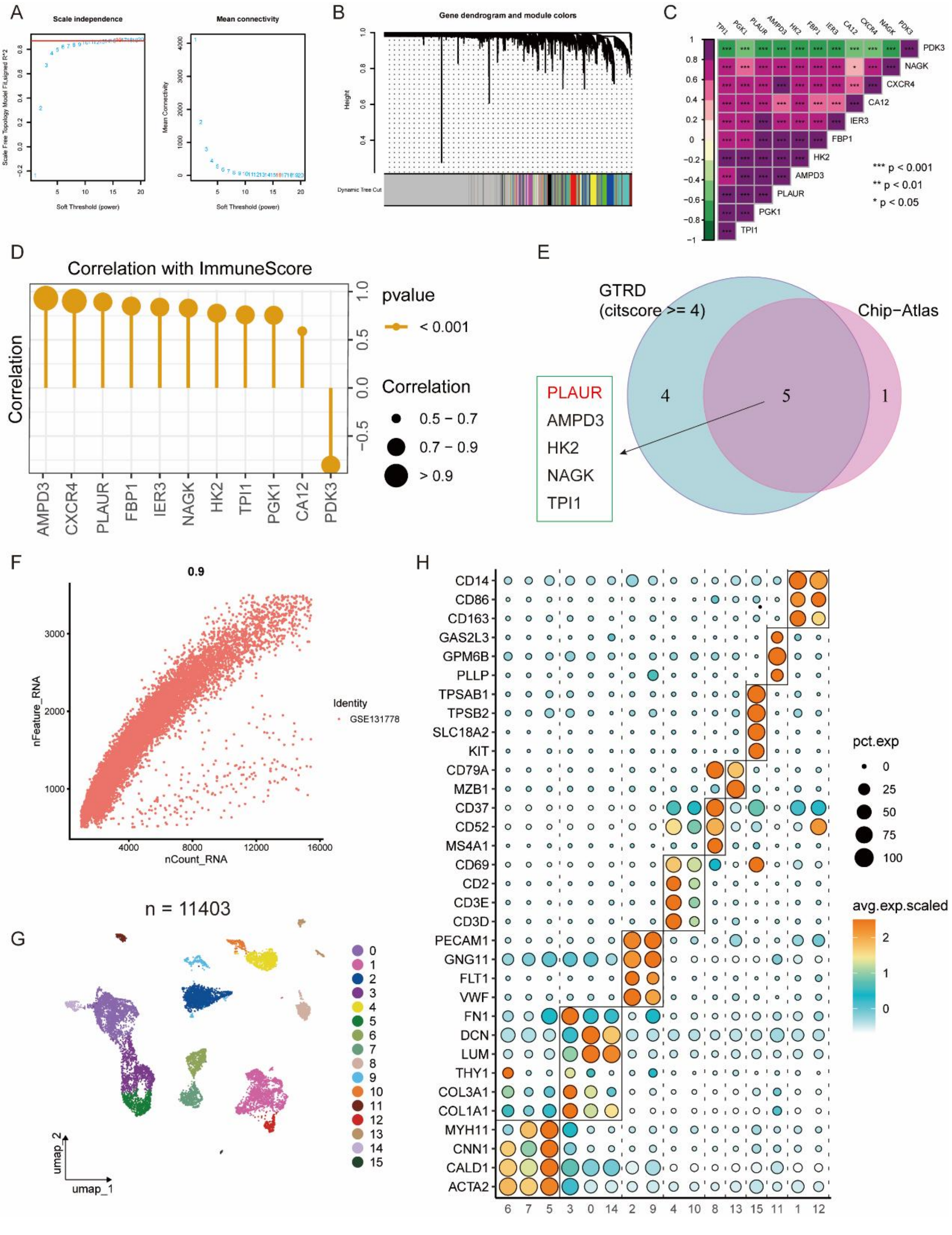

Supplementary Figure S2. Validation and selection of AA-specific candidate genes involved in hypoxia. (A) Soft threshold selection process. (B) Cluster dendrogram. Each color represents one specific co-expression module, and the colored rows below the dendrogram represent the merged modules. (C) Heatmap showing the correlation between 11 candidate genes. (D) Lollipop plot showing the correlation between ImmuneScore and candidate genes. The size of the ball indicates the magnitude of the correlation and different colors indicate different p values. (E) Venn plot showing 5 candidate genes predicted by both the database GTRD and Chip-Atlas as potential target

genes for HIF1A. (F) Scatter plot showing data quality of GSE131778. (G) UMAP projections of 11403 single cells from 4 cases were shown to form 16 major clusters. (H) Dot plots showing the average expression of known markers in the cell types depicted.

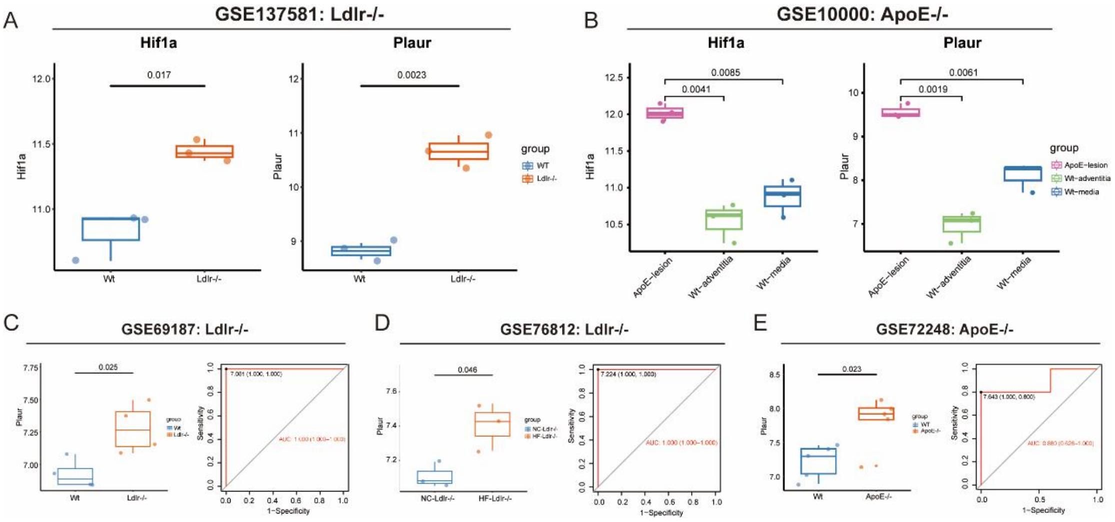

Supplementary Figure S3. External Validation of Plaur as a Valid Diagnostic Marker of AS in Mice. (A) Hif1a and Plaur were considerably elevated in AS plaques in Ldlr-/- mice (p = 0.017, p = 0.0023, respectively). (B) Boxplots showing the expression of Hif1a and Plaur in mouse AS lesions was significantly elevated in ApoE-/- model mice compared to the outer and inner membranes of Wt mice. (C) Plaur was greatly increased in AS plaques in Ldlr-/- mice (p = 0.025); ROC curves of Plaur in GSE69187 dataset, AUC = 1.000 (95%CI: 1.000-1.000). (D) Plaur were significantly elevated in AS plaques in Ldlr-/- mice (p = 0.046); ROC curves of Plaur in GSE76812 dataset, AUC = 1.000 (95%CI: 1.000-1.000). (E) Plaur was significantly increased in AS plaques in ApoE-/- mice (p = 0.023); ROC curves of Plaur in GSE72248 dataset, AUC = 0.880 (95%CI: 0.626-1.000).

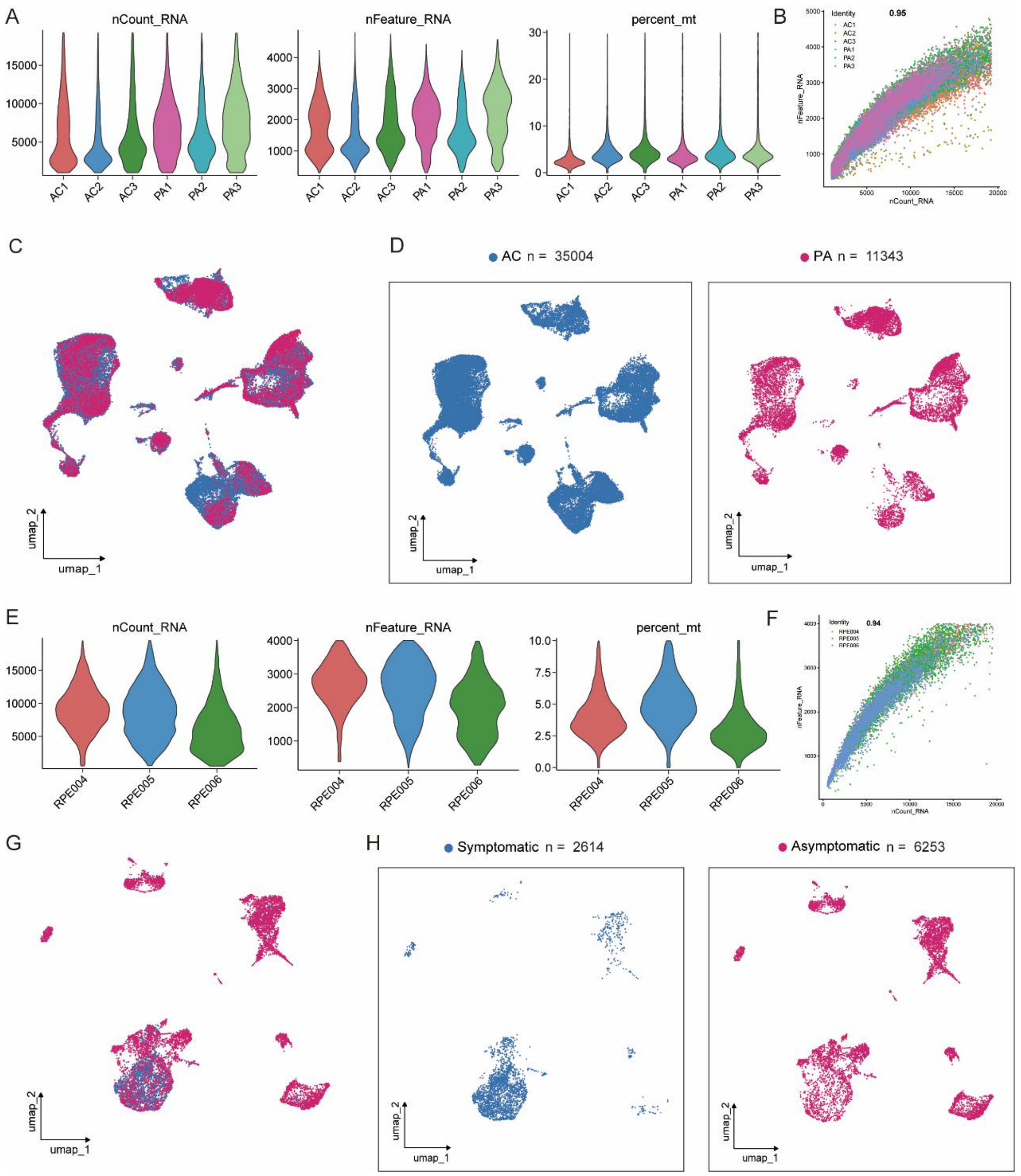

Supplementary Figure S4. External Validation of PLAUR As A Valid Diagnostic Marker of AS in Human. (A) Violin plot showing detected gene number, transcript number and mitochondrial gene percentage of each sample. (B) Scatter plot showing data quality of GSE159677. (C) UMAP plot showing the different cell types (n = 46347) of the scRNA-seq dataset GSE159677 with pathological information. (D) Pathological information included 3 AS cores (AC, n = 35004) and 3 patient-matched proximal adjacents (PA, n = 11343). (E) Violin plot showing detected gene number, transcript number and mitochondrial gene percentage of each sample. (F) Scatter plot showing data quality of GSE155512. (G) UMAP plot showing the different cell types (n = 8867) of the scRNA-seq dataset GSE15512 with pathological information. (H) Pathological information includes a patient with symptomatic (n = 2614) and 2 patients with asymptomatic (n = 6253).

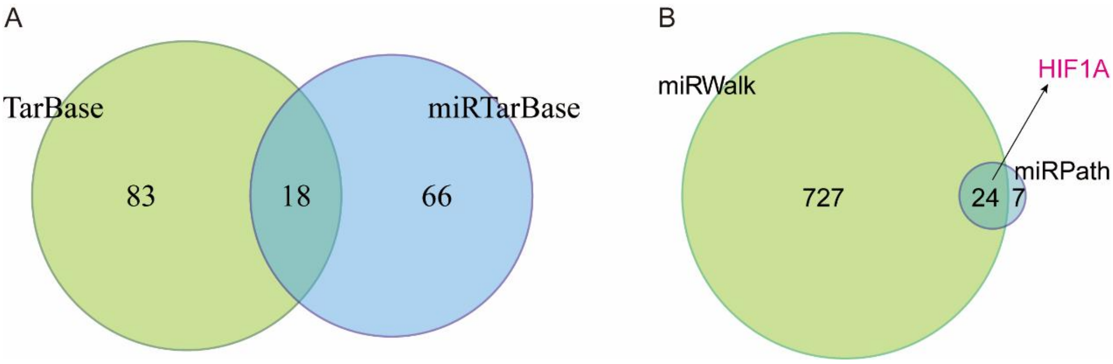

Supplementary Figure S5. Construction and Verification of the ceRNA Network that Regulates HIF1A. (A) Venn plot showing 18 overlapping results predicted by both TarBase and miRTarBase as possible target miRNA for HIF1A. (B) Venn plot showing a total of 24 overlapping results predicted by both the databases miRWalk and miRPath as possible target genes for has-miR-424-5p.

Supplementary Tables

Supplementary Table1: Results of using HumanTFDB to predict transcription factors of PLAUR (only the part of transcription factor HIF1A is shown).

TF	Start	Stop	Strand	Score	P-value	Q-value	Matched Sequence
HIF1A	1895	1910	-	14.1429	6.44e-06	0.0238	CCTCCCCCTCCTCCCG
HIF1A	455	473	+	11.8289	1.05e-05	0.0412	CTGGGTGACAGAGTGAGAC
HIF1A	1749	1764	-	11.9481	3.61e-05	0.0667	GTTCTCTCTCTTCCTA
HIF1A	339	354	-	11.1299	5.27e-05	0.207	CACGCCAGCACACCCG
HIF1A	453	470	-	2.22368	5.55e-05	0.212	TCACTCTGTCACCCAGGC
HIF1A	1008	1019	+	11.3506	5.63e-05	0.214	AAAAGAAAAAGA
HIF1A	1898	1913	-	10.8571	7.6e-05	0.0935	GCGCCTCCCCCTCCTC

Supplementary Table2: Results of using GTRD to predict transcription factors of PLAUR (only the part of transcription factor HIF1A is shown).

Chromosome	From	To	TF	ID
chr19	43652458	43652540	HIF1A	ms.HIF1A_HUMAN.168784.v1
chr19	43668532	43668604	HIF1A	ms.HIF1A_HUMAN.168784.v1
chr19	43668807	43668862	HIF1A	ms.HIF1A_HUMAN.168784.v1
chr19	43668924	43668996	HIF1A	ms.HIF1A_HUMAN.168784.v1
chr19	43669014	43669086	HIF1A	ms.HIF1A_HUMAN.168784.v1

chr19	43669874	43669956	HIF1A	ms.HIF1A_HUMAN.168784.v1
chr19	43670126	43670198	HIF1A	ms.HIF1A_HUMAN.168784.v1
chr19	43670261	43670332	HIF1A	ms.HIF1A_HUMAN.168784.v1
chr19	43674746	43674818	HIF1A	ms.HIF1A_HUMAN.168784.v1
chr19	43674831	43674903	HIF1A	ms.HIF1A_HUMAN.168784.v1

Supplementary Table3: Results of using JASPAR to predict transcription factors of PLAUR (only the part of transcription factor HIF1A is shown).

TF	Score	Relative Score	Start	Stop	Strand	Prediction Sequence
HIF1A	7.368294	0.88571958656013	1914	1921	-	ATGCGTGG
HIF1A	5.8164825	0.8394252645983201	1495	1502	+	CTTCGTGC
HIF1A	5.70392	0.8360672454043262	102	109	-	GTAGGTGC
HIF1A	5.70392	0.8360672454043262	1418	1425	+	GTAGGTGC
HIF1A	5.6903157	0.8356613999338561	795	802	+	GTACGGGC
HIF1A	5.204789	0.8211769557530465	348	355	+	TGGCGTGC
HIF1A	5.1526313	0.8196209587289864	1266	1273	-	GATCGTGC
HIF1A	4.860436	0.810904058073405	203	210	+	CCAGGTGC
HIF1A	4.8468323	0.8104982268281529	252	259	-	CCACCTGC
HIF1A	4.6482997	0.8045755146246252	340	347	+	GGGTGTGC

Supplementary Table4: Prediction results of miRNA targeting HIF1A based on the TarBase dataset and miRTarBase dataset.

	mature_mirna_id	target_symbol	mirtarbase	tarbase	validated.sum	all.sum
1	hsa-miR-217	HIF1A	1	1	2	2
2	hsa-miR-17-5p	HIF1A	3	1	2	2
3	hsa-miR-18a-5p	HIF1A	5	1	2	2
4	hsa-miR-27a-3p	HIF1A	1	1	2	2
5	hsa-miR-93-5p	HIF1A	3	1	2	2
6	hsa-miR-107	HIF1A	1	1	2	2
7	hsa-miR-210-3p	HIF1A	1	1	2	2
8	hsa-miR-138-5p	HIF1A	2	1	2	2
9	hsa-miR-142-5p	HIF1A	1	1	2	2
10	hsa-miR-186-5p	HIF1A	1	1	2	2
11	hsa-miR-155-5p	HIF1A	1	1	2	2
12	hsa-miR-338-3p	HIF1A	1	1	2	2
13	hsa-miR-424-5p	HIF1A	1	1	2	2

14	hsa-miR-429	HIF1A	1	1	2	2
15	hsa-miR-494-3p	HIF1A	1	1	2	2
16	hsa-miR-625-3p	HIF1A	1	1	2	2
17	hsa-miR-2682-5p	HIF1A	1	1	2	2
18	hsa-miR-4487	HIF1A	1	1	2	2
19	hsa-miR-17-92	HIF1A	0	0	1	1
20	hsa-miR-375	HIF1A	0	1	1	1
21	hsa-let-7b-5p	HIF1A	1	0	1	1
22	hsa-let-7d-5p	HIF1A	0	1	1	1
23	hsa-miR-16-5p	HIF1A	0	1	1	1
24	hsa-miR-19a-3p	HIF1A	0	1	1	1
25	hsa-miR-19b-3p	HIF1A	0	1	1	1
26	hsa-miR-20a-5p	HIF1A	3	0	1	1
27	hsa-miR-21-5p	HIF1A	1	0	1	1
28	hsa-miR-22-3p	HIF1A	1	0	1	1
29	hsa-miR-23a-3p	HIF1A	0	1	1	1
30	hsa-miR-29a-3p	HIF1A	0	1	1	1
31	hsa-miR-31-5p	HIF1A	0	1	1	1
32	hsa-miR-33a-5p	HIF1A	2	0	1	1
33	hsa-miR-96-5p	HIF1A	0	1	1	1
34	hsa-miR-29b-3p	HIF1A	0	1	1	1
35	hsa-miR-106a-5p	HIF1A	1	0	1	1
36	hsa-miR-196a-5p	HIF1A	0	1	1	1
37	hsa-miR-199a-5p	HIF1A	6	0	1	1
38	hsa-miR-148a-3p	HIF1A	0	1	1	1
39	hsa-miR-10a-5p	HIF1A	0	1	1	1
40	hsa-miR-10b-5p	HIF1A	0	1	1	1
41	hsa-miR-34a-5p	HIF1A	0	1	1	1
42	hsa-miR-182-5p	HIF1A	0	1	1	1
43	hsa-miR-183-5p	HIF1A	0	1	1	1
44	hsa-miR-199b-5p	HIF1A	1	0	1	1
45	hsa-miR-218-5p	HIF1A	0	1	1	1
46	hsa-miR-1-3p	HIF1A	0	1	1	1
47	hsa-miR-23b-3p	HIF1A	0	2	1	1
48	hsa-miR-27b-3p	HIF1A	0	1	1	1
49	hsa-miR-124-3p	HIF1A	0	1	1	1
50	hsa-miR-128-3p	HIF1A	0	1	1	1
51	hsa-miR-130a-3p	HIF1A	0	1	1	1

52	hsa-miR-133a-3p	HIF1A	0	1	1	1
53	hsa-miR-140-5p	HIF1A	0	1	1	1
54	hsa-miR-145-5p	HIF1A	1	0	1	1
55	hsa-miR-9-5p	HIF1A	0	1	1	1
56	hsa-miR-193a-3p	HIF1A	0	1	1	1
57	hsa-miR-195-5p	HIF1A	0	1	1	1
58	hsa-miR-106b-5p	HIF1A	2	0	1	1
59	hsa-miR-29c-3p	HIF1A	0	1	1	1
60	hsa-miR-34b-5p	HIF1A	1	0	1	1
61	hsa-miR-299-3p	HIF1A	0	1	1	1
62	hsa-miR-376c-3p	HIF1A	0	1	1	1
63	hsa-miR-370-3p	HIF1A	0	1	1	1
64	hsa-miR-373-3p	HIF1A	0	1	1	1
65	hsa-miR-374a-5p	HIF1A	0	1	1	1
66	hsa-miR-376a-3p	HIF1A	0	1	1	1
67	hsa-miR-377-3p	HIF1A	0	1	1	1
68	hsa-miR-382-5p	HIF1A	0	1	1	1
69	hsa-miR-151a-3p	HIF1A	1	0	1	1
70	hsa-miR-148b-3p	HIF1A	0	1	1	1
71	hsa-miR-324-5p	HIF1A	0	1	1	1
72	hsa-miR-325	HIF1A	0	1	1	1
73	hsa-miR-196b-5p	HIF1A	0	1	1	1
74	hsa-miR-18b-5p	HIF1A	3	0	1	1
75	hsa-miR-20b-5p	HIF1A	4	0	1	1
76	hsa-miR-433-3p	HIF1A	1	0	1	1
77	hsa-miR-412-3p	HIF1A	0	1	1	1
78	hsa-miR-410-3p	HIF1A	0	1	1	1
79	hsa-miR-376b-3p	HIF1A	0	1	1	1
80	hsa-miR-485-5p	HIF1A	0	1	1	1
81	hsa-miR-193b-3p	HIF1A	0	1	1	1
82	hsa-miR-519c-3p	HIF1A	1	0	1	1
83	hsa-miR-518c-5p	HIF1A	0	1	1	1
84	hsa-miR-519d-3p	HIF1A	1	0	1	1
85	hsa-miR-299-5p	HIF1A	1	0	1	1
86	hsa-miR-558	HIF1A	1	0	1	1
87	hsa-miR-575	HIF1A	0	1	1	1
88	hsa-miR-580-3p	HIF1A	0	1	1	1
89	hsa-miR-583	HIF1A	0	1	1	1

90	hsa-miR-587	HIF1A	0	1	1	1
91	hsa-miR-589-3p	HIF1A	1	0	1	1
92	hsa-miR-616-5p	HIF1A	0	1	1	1
93	hsa-miR-647	HIF1A	0	1	1	1
94	hsa-miR-548d-3p	HIF1A	0	1	1	1
95	hsa-miR-659-3p	HIF1A	0	1	1	1
96	hsa-miR-16-1-3p	HIF1A	0	1	1	1
97	hsa-miR-22-5p	HIF1A	0	1	1	1
98	hsa-miR-27a-5p	HIF1A	0	1	1	1
99	hsa-miR-29a-5p	HIF1A	0	1	1	1
100	hsa-miR-32-3p	HIF1A	0	1	1	1
101	hsa-miR-93-3p	HIF1A	0	1	1	1
102	hsa-miR-100-3p	HIF1A	0	1	1	1
103	hsa-miR-106a-3p	HIF1A	1	0	1	1
104	hsa-miR-16-2-3p	HIF1A	0	1	1	1
105	hsa-miR-10b-3p	HIF1A	0	1	1	1
106	hsa-miR-23b-5p	HIF1A	0	1	1	1
107	hsa-miR-138-1-3p	HIF1A	1	0	1	1
108	hsa-miR-146a-3p	HIF1A	0	1	1	1
109	hsa-miR-200c-5p	HIF1A	1	0	1	1
110	hsa-miR-106b-3p	HIF1A	1	0	1	1
111	hsa-miR-342-5p	HIF1A	0	1	1	1
112	hsa-miR-337-5p	HIF1A	0	1	1	1
113	hsa-miR-497-3p	HIF1A	1	0	1	1
114	hsa-miR-501-3p	HIF1A	0	1	1	1
115	hsa-miR-502-3p	HIF1A	0	1	1	1
116	hsa-miR-509-5p	HIF1A	1	0	1	1
117	hsa-miR-576-3p	HIF1A	1	0	1	1
118	hsa-miR-590-3p	HIF1A	0	1	1	1
119	hsa-miR-889-3p	HIF1A	1	0	1	1
120	hsa-miR-509-3-5p	HIF1A	1	0	1	1
121	hsa-miR-935	HIF1A	1	0	1	1
122	hsa-miR-940	HIF1A	1	0	1	1
123	hsa-miR-1260a	HIF1A	0	1	1	1
124	hsa-miR-548o-3p	HIF1A	0	1	1	1
125	hsa-miR-1276	HIF1A	0	1	1	1
126	hsa-miR-664a-3p	HIF1A	0	1	1	1
127	hsa-miR-1322	HIF1A	2	0	1	1

128	hsa-miR-449c-5p	HIF1A	1	0	1	1
129	hsa-miR-3121-3p	HIF1A	1	0	1	1
130	hsa-miR-3128	HIF1A	1	0	1	1
131	hsa-miR-3074-3p	HIF1A	1	0	1	1
132	hsa-miR-3160-3p	HIF1A	1	0	1	1
133	hsa-miR-1260b	HIF1A	0	1	1	1
134	hsa-miR-4282	HIF1A	1	0	1	1
135	hsa-miR-3609	HIF1A	1	0	1	1
136	hsa-miR-3613-3p	HIF1A	0	1	1	1
137	hsa-miR-23c	HIF1A	0	1	1	1
138	hsa-miR-3662	HIF1A	2	0	1	1
139	hsa-miR-3668	HIF1A	1	0	1	1
140	hsa-miR-3909	HIF1A	0	1	1	1
141	hsa-miR-548z	HIF1A	0	1	1	1
142	hsa-miR-4418	HIF1A	1	0	1	1
143	hsa-miR-548ah-5p	HIF1A	1	0	1	1
144	hsa-miR-4463	HIF1A	1	0	1	1
145	hsa-miR-4464	HIF1A	1	0	1	1
146	hsa-miR-4470	HIF1A	1	0	1	1
147	hsa-miR-4499	HIF1A	1	0	1	1
148	hsa-miR-4662a-5p	HIF1A	2	0	1	1
149	hsa-miR-4735-3p	HIF1A	2	0	1	1
150	hsa-miR-4748	HIF1A	1	0	1	1
151	hsa-miR-4799-5p	HIF1A	1	0	1	1
152	hsa-miR-5094	HIF1A	1	0	1	1
153	hsa-miR-5681b	HIF1A	1	0	1	1
154	hsa-miR-5692a	HIF1A	2	0	1	1
155	hsa-miR-548h-3p	HIF1A	0	1	1	1
156	hsa-miR-1277-5p	HIF1A	0	1	1	1
157	hsa-miR-5089-3p	HIF1A	2	0	1	1
158	hsa-miR-6511a-5p	HIF1A	1	0	1	1
159	hsa-miR-153-5p	HIF1A	3	0	1	1
160	hsa-miR-329-5p	HIF1A	1	0	1	1
161	hsa-miR-1910-3p	HIF1A	1	0	1	1
162	hsa-miR-6755-5p	HIF1A	1	0	1	1
163	hsa-miR-6806-5p	HIF1A	1	0	1	1
164	hsa-miR-6807-3p	HIF1A	1	0	1	1
165	hsa-miR-6808-5p	HIF1A	1	0	1	1

166	hsa-miR-6815-3p	HIF1A	1	0	1	1
167	hsa-miR-6893-5p	HIF1A	1	0	1	1
168	hsa-miR-8055	HIF1A	1	0	1	1

Supplementary Table5: Prediction results of target genes of hsa-miR-424-5p based on the miRWalk dataset (only predictions with experimental support were retained).

genesymbol	bindingp	energy	validated
GPR180	0.807692308	-17.6	MIRT190188
TBPL1	0.807692308	-26.2	MIRT042456
ORC4	0.807692308	-19.3	MIRT498980
PLAG1	0.833333333	-17.7	MIRT000696
SLC39A9	0.833333333	-16.5	MIRT191629
CDK6	0.846153846	-19.1	MIRT000938
CDK6	0.846153846	-18	MIRT000938
GNAL	0.846153846	-18	MIRT476273
GNAL	0.846153846	-18	MIRT476273
ZBTB10	0.846153846	-17.9	MIRT545959
ACVR2A	0.846153846	-18.5	MIRT568625
ACVR2A	0.846153846	-18.5	MIRT568625
OSCAR	0.846153846	-15.4	MIRT550466
OSCAR	0.846153846	-15.4	MIRT550466
PDIA6	0.846153846	-15.8	MIRT574539
PDIA6	0.846153846	-15.8	MIRT574539
RNPS1	0.846153846	-17.1	MIRT743308
ANKRD17	0.846153846	-19.3	MIRT042449
CCND3	0.846153846	-17.9	MIRT000937
CCND3	0.846153846	-17.9	MIRT000937
TTC39A	0.846153846	-17.4	MIRT042435
LAMTOR4	0.846153846	-15.7	MIRT042451
ANKRD17	0.846153846	-19.3	MIRT042449
DYNLL2	0.846153846	-17.9	MIRT251497
PTPRD	0.846153846	-17.6	MIRT555142
PTPRD	0.846153846	-17.6	MIRT555142
OSCAR	0.846153846	-15.4	MIRT550466
OSCAR	0.846153846	-15.4	MIRT550466
OSCAR	0.846153846	-15.4	MIRT550466
MAP3K7	0.846153846	-18.6	MIRT566831
MAP3K7	0.846153846	-18.6	MIRT566831

MAP3K7	0.846153846	-18.6	MIRT566831
TPM3	0.846153846	-20.7	MIRT553439
ZNF449	0.846153846	-19.4	MIRT229350
SNX16	0.846153846	-15.6	MIRT505546
APBB2	0.846153846	-17.5	MIRT042448
LSM11	0.846153846	-24.5	MIRT571928
HIF1A	0.846153846	-15.8	MIRT005926
PIK3R1	0.846153846	-15.1	MIRT314055
ORC4	0.846153846	-17.9	MIRT498980
ORC4	0.846153846	-19.3	MIRT498980
ORC4	0.846153846	-17.9	MIRT498980
ANKRD17	0.846153846	-19.3	MIRT042449
RBPJ	0.846153846	-22.1	MIRT212611
RBPJ	0.846153846	-22.1	MIRT212611
OSCAR	0.846153846	-15.4	MIRT550466
RAPH1	0.846153846	-20.8	MIRT563896
RAPH1	0.846153846	-20.8	MIRT563896
ATF6	0.846153846	-17.7	MIRT000658
ATF6	0.846153846	-17.7	MIRT000658
MAP2K3	0.846153846	-17.5	MIRT743304
APBB2	0.846153846	-17.5	MIRT042448
CALU	0.846153846	-16.2	MIRT102441
FGFR1	0.846153846	-18.4	MIRT003228
FGFR1	0.846153846	-17.1	MIRT003228
FGFR1	0.846153846	-18.4	MIRT003228
FGFR1	0.846153846	-17.1	MIRT003228
MAP2K3	0.846153846	-17.5	MIRT743304
SOCS6	0.846153846	-17.5	MIRT053149
PISD	0.846153846	-19.6	MIRT471035
EPM2AIP1	0.846153846	-17.9	MIRT551379
FGFR1	0.846153846	-18.4	MIRT003228
FGFR1	0.846153846	-17.6	MIRT003228
RBPJ	0.846153846	-22.1	MIRT212611
TM7SF3	0.846153846	-19.7	MIRT553618
BSPRY	0.846153846	-18.9	MIRT645510
SBNO1	0.846153846	-18.5	MIRT521203
PAG1	0.846153846	-18.6	MIRT547234
ODF2L	0.846153846	-17	MIRT499452

DMTF1	0.846153846	-15.7	MIRT104236
FGFR1	0.846153846	-18.4	MIRT003228
FGFR1	0.846153846	-17.6	MIRT003228
FGFR1	0.846153846	-18.4	MIRT003228
FGFR1	0.846153846	-17.6	MIRT003228
FGFR1	0.846153846	-18.4	MIRT003228
ATG9A	0.846153846	-17.9	MIRT503143
ATG9A	0.846153846	-17.1	MIRT503143
MAP2K3	0.846153846	-17.5	MIRT743304
MAP3K7	0.846153846	-18.2	MIRT566831
TGFBR3	0.846153846	-17.3	MIRT565401
CUL3	0.846153846	-19.2	MIRT659256
CUL2	0.846153846	-19.3	MIRT005927
SMARCA5	0.846153846	-19.8	MIRT042457
PLPP3	0.846153846	-17.8	MIRT555432
FASN	0.846153846	-19.6	MIRT053106
SOCS6	0.846153846	-18.3	MIRT053149
RBPJ	0.846153846	-22.1	MIRT212611
MYB	0.846153846	-17.4	MIRT005886
AKT3	0.846153846	-17.5	MIRT482117
PDIA6	0.846153846	-17.6	MIRT574539
LANCL1	0.846153846	-18.4	MIRT562027
IVNS1ABP	0.846153846	-18.1	MIRT475065
SNTB2	0.846153846	-18.3	MIRT075256
SRSF1	0.846153846	-16.8	MIRT505502
ATF6	0.846153846	-21.7	MIRT000658
NCKAP1	0.846153846	-18.6	MIRT566650
CD274	0.846153846	-17.4	MIRT767955
PISD	0.846153846	-19.5	MIRT471035
LUC7L3	0.846153846	-18.7	MIRT449186
APBB2	0.846153846	-17.5	MIRT042448
FGFR1	0.846153846	-18.4	MIRT003228
FGFR1	0.846153846	-18.4	MIRT003228
FGFR1	0.846153846	-17.1	MIRT003228
FGFR1	0.846153846	-18.4	MIRT003228
RBPJ	0.846153846	-22.1	MIRT212611
TPM3	0.846153846	-20.7	MIRT553439
GNAL	0.846153846	-18	MIRT476273

RBPJ	0.846153846	-22.1	MIRT212611
RBPJ	0.846153846	-22.1	MIRT212611
ACVR2A	0.846153846	-18.5	MIRT568625
CCND3	0.846153846	-17.9	MIRT000937
ENTPD1	0.846153846	-19.5	MIRT127740
FKBP1A	0.846153846	-17.3	MIRT548223
CLEC2D	0.846153846	-18.4	MIRT574202
ODF2L	0.846153846	-17	MIRT499452
LAMTOR4	0.846153846	-15.7	MIRT042451
TFAP2A	0.846153846	-14.9	MIRT466433
PTPRD	0.846153846	-17.6	MIRT555142
ATG9A	0.846153846	-17.9	MIRT503143
SRSF1	0.846153846	-16.8	MIRT505502
ENTPD1	0.846153846	-19.5	MIRT127740
HIGD1A	0.846153846	-17.6	MIRT547970
ZBTB10	0.846153846	-17.9	MIRT545959
MYB	0.846153846	-17.4	MIRT005886
MYB	0.846153846	-17.4	MIRT005886
CALU	0.846153846	-15.5	MIRT102441
CCND3	0.846153846	-18.3	MIRT000937
CPSF7	0.846153846	-17.7	MIRT478908
CCND3	0.846153846	-17.9	MIRT000937
CCND3	0.846153846	-18.3	MIRT000937
LANCL1	0.846153846	-18.4	MIRT562027
DMTF1	0.846153846	-15.7	MIRT104236
DMTF1	0.846153846	-15.7	MIRT104236
GNAL	0.846153846	-18	MIRT476273
WEE1	0.846153846	-16.6	MIRT000659
CDK6	0.846153846	-19.1	MIRT000938
CDK6	0.846153846	-18	MIRT000938
MYB	0.846153846	-17.4	MIRT005886
MYB	0.846153846	-17.4	MIRT005886
MYB	0.846153846	-17.4	MIRT005886
MYB	0.846153846	-17.4	MIRT005886
MYB	0.846153846	-17.4	MIRT005886
ENTPD1	0.846153846	-19.5	MIRT127740
ENTPD1	0.846153846	-19.5	MIRT127740
APBB2	0.846153846	-17.5	MIRT042448

APBB2	0.846153846	-16.1	MIRT042448
APBB2	0.846153846	-16.1	MIRT042448
SBNO1	0.846153846	-18.5	MIRT521203
FGFR1	0.846153846	-18.4	MIRT003228
FGFR1	0.846153846	-18.4	MIRT003228
FGFR1	0.846153846	-18.4	MIRT003228
CLSPN	0.846153846	-17.9	MIRT544912
CLSPN	0.846153846	-17.7	MIRT544912
SMAD7	0.846153846	-21.5	MIRT501088
ORC4	0.846153846	-19.3	MIRT498980
ORC4	0.846153846	-17.9	MIRT498980
ORC4	0.846153846	-19.3	MIRT498980
ORC4	0.846153846	-17.9	MIRT498980
CUL2	0.846153846	-19.3	MIRT005927
CUL2	0.846153846	-19.3	MIRT005927
CALU	0.846153846	-15.5	MIRT102441
CALU	0.846153846	-15.5	MIRT102441
CALU	0.846153846	-15.5	MIRT102441
FKBP1A	0.846153846	-19.9	MIRT548223
CALU	0.846153846	-15.5	MIRT102441
ANAPC13	0.846153846	-15.8	MIRT543408
ANAPC13	0.846153846	-15.8	MIRT543408
SLC2A3	0.871794872	-18.7	MIRT521056
ANKMY1	0.871794872	-17.1	MIRT618896
ANKMY1	0.871794872	-17.1	MIRT618896
ANKMY1	0.871794872	-17.1	MIRT618896
PTCH1	0.884615385	-20.6	MIRT438750
PTCH1	0.884615385	-20.6	MIRT438750
PTCH1	0.884615385	-20.6	MIRT438750
CCNT1	0.884615385	-19.7	MIRT564332
MAP2K1	0.897435897	-22.4	MIRT003226
MAP2K1	0.897435897	-22.4	MIRT003226
CUL3	0.923076923	-19.2	MIRT659256
CUL3	0.923076923	-19.2	MIRT659256
UBE2V1	0.923076923	-15.3	MIRT464666
UBE2V1	0.923076923	-15.3	MIRT464666
SOCS2	0.923076923	-17.6	MIRT731524
DNAJC10	0.923076923	-19.3	MIRT552035

CCNT1	0.923076923	-18.7	MIRT564332
UBE2V1	0.923076923	-15.3	MIRT464666
UBE2V1	0.923076923	-15.3	MIRT464666
RNPS1	0.923076923	-17.1	MIRT743308
RNPS1	0.923076923	-17.1	MIRT743308
CHIC1	0.923076923	-16.2	MIRT327967
C11orf24	0.923076923	-18	MIRT549121
KIAA0895	0.923076923	-19	MIRT497772
KIAA0895	0.923076923	-17.4	MIRT497772
MAP4K2	0.923076923	-18.3	MIRT545186
BSPRY	0.923076923	-18.9	MIRT645510
PIAS1	0.923076923	-16.3	MIRT003227
SOCS5	0.923076923	-16.4	MIRT302829
CDCA4	0.923076923	-16.1	MIRT502919
ZMAT3	0.923076923	-15.4	MIRT500422
CNKSR3	0.923076923	-18.8	MIRT558662
PPM1A	0.923076923	-22.6	MIRT191460
GNAL	0.923076923	-18	MIRT476273
PEDS1	0.923076923	-18.6	MIRT466009
HNRNPA2B1	0.923076923	-18.4	MIRT562201
GLP2R	0.923076923	-16.3	MIRT682514
ATF6	0.923076923	-21.7	MIRT000658
ATF6	0.923076923	-21.7	MIRT000658
CCND3	0.923076923	-15.3	MIRT000937
SIAH1	0.923076923	-19.6	MIRT005887
CLUH	0.923076923	-19.8	MIRT743296
CANX	0.923076923	-19.4	MIRT096239
CPSF7	0.923076923	-19.4	MIRT478908
AKT3	0.923076923	-17.5	MIRT482117
HNRNPA2B1	0.923076923	-18.4	MIRT562201
CPSF7	0.923076923	-19.4	MIRT478908
WNK3	0.923076923	-20.2	MIRT564989
WNK3	0.923076923	-20.2	MIRT564989
CBX6	0.923076923	-15.7	MIRT507882
CPEB3	0.923076923	-17	MIRT512642
KIAA0895	0.923076923	-17.4	MIRT497772
ANAPC13	0.923076923	-15.8	MIRT543408
TOB2	0.923076923	-18	MIRT465566

LUC7L3	0.923076923	-18.9	MIRT449186
CDCA4	0.923076923	-16.7	MIRT502919
ASH1L	0.923076923	-18.4	MIRT549276
DNAJC10	0.923076923	-19.3	MIRT552035
TRMT9B	0.923076923	-16.7	MIRT562877
PPM1A	0.923076923	-17.5	MIRT191460
RECK	0.923076923	-18.4	MIRT107689
PPP1R11	0.923076923	-15	MIRT100211
ZMAT3	0.923076923	-15.4	MIRT500422
FGFR1	0.923076923	-17.1	MIRT003228
ZBTB10	0.923076923	-17.5	MIRT545959
RAD23B	0.923076923	-17.5	MIRT106737
SIAH1	0.923076923	-19.6	MIRT005887
SKI	0.923076923	-17.2	MIRT133776
WEE1	0.923076923	-16.6	MIRT000659
SMARCA5	0.923076923	-24.3	MIRT042457
GPRC5A	0.923076923	-19.9	MIRT621498
APBB2	0.923076923	-18	MIRT042448
MAP4K2	0.923076923	-18.3	MIRT545186
ABL2	0.923076923	-19	MIRT482553
CRKL	0.923076923	-17.7	MIRT367269
CD180	0.923076923	-16.8	MIRT496015
SMAD3	0.923076923	-19.4	MIRT733535
PDE4D	0.923076923	-18.1	MIRT471492
NUP50	0.923076923	-16.1	MIRT206023
ATF6	0.923076923	-17.7	MIRT000658
SHOC2	0.923076923	-18	MIRT055427
CLEC2D	0.923076923	-17.8	MIRT574202
SEC61A1	0.923076923	-17.2	MIRT743310
SOCS5	0.923076923	-16.4	MIRT302829
PDE4D	0.923076923	-18.1	MIRT471492
CANX	0.923076923	-19.4	MIRT096239
CANX	0.923076923	-19.4	MIRT096239
CANX	0.923076923	-19.4	MIRT096239
CANX	0.923076923	-19.4	MIRT096239
CANX	0.923076923	-19.4	MIRT096239
CANX	0.923076923	-19.4	MIRT096239
CANX	0.923076923	-19.4	MIRT096239

CLUH	0.923076923	-19.8	MIRT743296
CLUH	0.923076923	-19.8	MIRT743296
ZNF275	0.923076923	-16.9	MIRT109245
AKT3	0.923076923	-17.5	MIRT482117
EIF1AX	0.923076923	-19	MIRT477561
CANX	0.923076923	-19.4	MIRT096239
ENTPD1	0.923076923	-21.1	MIRT127740
PTCH1	0.923076923	-20.6	MIRT438750
WNK3	0.923076923	-20.2	MIRT564989
CLEC2D	0.923076923	-17.8	MIRT574202
SIAH1	0.923076923	-19.6	MIRT005887
CANX	0.923076923	-19.4	MIRT096239
UBE2V1	0.923076923	-15.3	MIRT464666
EIF4G2	0.923076923	-17.9	MIRT042446
ATG9A	0.923076923	-17.1	MIRT503143
ENTPD1	0.923076923	-21.1	MIRT127740
KIAA0895	0.923076923	-18.3	MIRT497772
PDE4D	0.923076923	-18.1	MIRT471492
ZBTB10	0.923076923	-17.5	MIRT545959
AMOT	0.923076923	-17.8	MIRT549390
PLAG1	0.923076923	-17.7	MIRT000696
PLAG1	0.923076923	-17.7	MIRT000696
CALU	0.923076923	-16.2	MIRT102441
ABL2	0.923076923	-19	MIRT482553
CPSF7	0.923076923	-19.4	MIRT478908
LANCL1	0.923076923	-18.4	MIRT562027
CPSF7	0.923076923	-17.7	MIRT478908
SMAD3	0.923076923	-19.4	MIRT733535
SMAD3	0.923076923	-19.4	MIRT733535
SMAD3	0.923076923	-19.4	MIRT733535
PEDS1	0.923076923	-18.6	MIRT466009
ENTPD1	0.923076923	-21.1	MIRT127740
ENTPD1	0.923076923	-21.1	MIRT127740
PDE4D	0.923076923	-18.1	MIRT471492
ABL2	0.923076923	-19	MIRT482553
ABL2	0.923076923	-19	MIRT482553
ABL2	0.923076923	-19	MIRT482553
ABL2	0.923076923	-19	MIRT482553

FGFR1	0.923076923	-17.1	MIRT003228
FGFR1	0.923076923	-17.1	MIRT003228
FGFR1	0.923076923	-17.1	MIRT003228
ARHGDIA	0.923076923	-19.1	MIRT512285
ORC4	0.923076923	-17.9	MIRT498980
TGFBR3	0.923076923	-17.3	MIRT565401
PDE4D	0.923076923	-18.1	MIRT471492
PDE4D	0.923076923	-18.1	MIRT471492
PDE4D	0.923076923	-18.1	MIRT471492
PDE4D	0.923076923	-18.1	MIRT471492
PDE4D	0.923076923	-18.1	MIRT471492
CLEC2D	0.923076923	-17.8	MIRT574202
CLEC2D	0.923076923	-17.8	MIRT574202
CLEC2D	0.923076923	-17.8	MIRT574202
CALU	0.923076923	-16.2	MIRT102441
CALU	0.923076923	-16.2	MIRT102441
CALU	0.923076923	-16.2	MIRT102441
KIAA0895	0.923076923	-18.3	MIRT497772
FKBP1A	0.923076923	-17.3	MIRT548223
CALU	0.923076923	-16.2	MIRT102441
CCNT1	0.923076923	-18.7	MIRT564332
PIK3R1	0.923076923	-15.1	MIRT314055
CUL3	1	-19.4	MIRT659256
CUL3	1	-19.1	MIRT659256
CUL3	1	-19.4	MIRT659256
CUL3	1	-19.1	MIRT659256
CDX2	1	-15.7	MIRT053379
SHOC2	1	-18	MIRT055427
CCNT1	1	-19.7	MIRT564332
PHLPP2	1	-17.2	MIRT547132
ARHGDIA	1	-19.1	MIRT512285
ARHGDIA	1	-19.1	MIRT512285
CBX6	1	-15.7	MIRT507882
TXNIP	1	-19.8	MIRT767959
RACGAP1	1	-18.2	MIRT554962
SCOC	1	-20.1	MIRT042453
CCNE2	1	-19.5	MIRT507850
CNKSR3	1	-20.6	MIRT558662

UBN2	1	-19.8	MIRT102636
ORC4	1	-18	MIRT498980
ORC4	1	-18	MIRT498980
CARM1	1	-19.5	MIRT767954
CDC25A	1	-18.6	MIRT000655
NCKAP1	1	-19.5	MIRT566650
PPIG	1	-20	MIRT506108
PPIG	1	-20	MIRT506108
CUL3	1	-19.9	MIRT659256
CUL2	1	-19.3	MIRT005927
RACGAP1	1	-18.2	MIRT554962
CCNE2	1	-19.5	MIRT507850
WNK3	1	-18.7	MIRT564989
WNK3	1	-18.7	MIRT564989
EIF2B2	1	-22.2	MIRT658793
HEYL	1	-22	MIRT475716
AP5Z1	1	-17.6	MIRT544589
PHLPP2	1	-17.2	MIRT547132
TOB2	1	-20.6	MIRT465566
AURKAIP1	1	-15.7	MIRT743292
ANLN	1	-18.7	MIRT000660
HOXA10	1	-16.9	MIRT541216
WNK3	1	-20.2	MIRT564989
WNK3	1	-18.7	MIRT564989
C11orf24	1	-18	MIRT549121
CPSF7	1	-19.4	MIRT478908
CPSF7	1	-17.7	MIRT478908
CDC25A	1	-18.6	MIRT000655
CLU	1	-18.9	MIRT743295
PIM1	1	-18.6	MIRT100572
CUL3	1	-19.4	MIRT659256
CUL3	1	-19.1	MIRT659256
GPRC5A	1	-17.7	MIRT621498
ARHGDIA	1	-19.1	MIRT512285
PPIG	1	-20	MIRT506108
CD180	1	-19.1	MIRT496015
CTDSPL	1	-18.6	MIRT307145
TXNIP	1	-19.8	MIRT767959

PLPBP	1	-24.7	MIRT574057
ABL2	1	-19	MIRT482553
CLEC2D	1	-18.4	MIRT574202
RACGAP1	1	-18.2	MIRT554962
CARM1	1	-19.5	MIRT767954
ENTPD1	1	-21.7	MIRT127740
EXT1	1	-17	MIRT558037
WNK3	1	-18.7	MIRT564989
CTDSPL	1	-18.6	MIRT307145
ENTPD1	1	-21.7	MIRT127740
ZBTB34	1	-20.4	MIRT107222
KIAA0895	1	-17.4	MIRT497772
RACGAP1	1	-18.2	MIRT554962
RACGAP1	1	-18.2	MIRT554962
AURKAIP1	1	-15.7	MIRT743292
AURKAIP1	1	-15.7	MIRT743292
ATXN7L3B	1	-18.8	MIRT248769
CPSF7	1	-19.4	MIRT478908
SCOC	1	-20.1	MIRT042453
SCOC	1	-20.1	MIRT042453
SCOC	1	-20.1	MIRT042453
SCOC	1	-20.1	MIRT042453
ENTPD1	1	-21.7	MIRT127740
ENTPD1	1	-21.7	MIRT127740
CPEB3	1	-17	MIRT512642
FBXL20	1	-16.9	MIRT548273
ARHGDIA	1	-19.1	MIRT512285
ORC4	1	-18	MIRT498980
ORC4	1	-18	MIRT498980
ORC4	1	-18	MIRT498980
TGFBR3	1	-17.3	MIRT565401
CLEC2D	1	-18.4	MIRT574202
CLEC2D	1	-18.4	MIRT574202
CLEC2D	1	-18.4	MIRT574202
CUL2	1	-19.3	MIRT005927
KIAA0895	1	-17.4	MIRT497772
KIAA0895	1	-18.3	MIRT497772
KIAA0895	1	-17.4	MIRT497772

KIAA0895	1	-18.3	MIRT497772
KIAA0895	1	-17.4	MIRT497772
RAD23B	1	-17.5	MIRT106737
SREK1	0.846153846	-16.9	MIRT500949
DCAF17	0.846153846	-21.1	MIRT680982
PHYHIP	0.846153846	-21.7	MIRT492711
RAB23	0.846153846	-17.7	MIRT555032
SLC39A9	0.846153846	-17.5	MIRT191629
CCND2	0.846153846	-17.1	MIRT064689
SLC39A9	0.846153846	-17.5	MIRT191629
SLC39A9	0.846153846	-17.5	MIRT191629
TPM3	0.923076923	-17.9	MIRT553439
BAZ2B	0.923076923	-16.6	MIRT042447
HSPA4L	0.923076923	-19.4	MIRT211317
NEGR1	0.923076923	-20	MIRT701506
PIK3R1	0.923076923	-17.6	MIRT314055
PIK3R1	0.923076923	-17.6	MIRT314055
ITPR1	0.923076923	-20	MIRT003225
ITPR1	0.923076923	-20	MIRT003225
ITPR1	0.923076923	-20	MIRT003225
ITPR1	0.923076923	-20	MIRT003225
OGT	0.923076923	-18.7	MIRT110057
ITPR1	0.923076923	-20	MIRT003225
RNPS1	0.923076923	-18.2	MIRT743308
BAZ2B	0.923076923	-16.6	MIRT042447
ITPR1	0.923076923	-20	MIRT003225
PHYHIP	0.923076923	-21.7	MIRT492711
ITPR1	0.923076923	-20	MIRT003225
ITPR1	0.923076923	-20	MIRT003225
UBE2V1	1	-19	MIRT464666
UBE2V1	1	-19	MIRT464666
UBE2V1	1	-19	MIRT464666
SLC2A3	1	-23.1	MIRT521056
SYNRG	0.807692308	-19.7	MIRT725132
SYNRG	0.807692308	-19.7	MIRT725132
NNT	0.807692308	-19	MIRT518994
EIF4G2	0.807692308	-16.5	MIRT042446
SYNRG	0.807692308	-19.7	MIRT725132

SYNRG	0.807692308	-19.7	MIRT725132
SYNRG	0.807692308	-19.7	MIRT725132
SEC24A	0.820512821	-16.6	MIRT468673
WIPI2	0.833333333	-25.2	MIRT258414
WIPI2	0.833333333	-25.2	MIRT258414
WIPI2	0.833333333	-25.2	MIRT258414
WIPI2	0.833333333	-25.2	MIRT258414
WIPI2	0.833333333	-25.2	MIRT258414
TRAK1	0.846153846	-18.1	MIRT614698
DNAJC10	0.846153846	-17.8	MIRT552035
AP2B1	0.846153846	-20.7	MIRT743291
AP2B1	0.846153846	-17	MIRT743291
FGFR4	0.846153846	-19.2	MIRT460220
MAP4K2	0.846153846	-18.3	MIRT545186
CCNE2	0.846153846	-20.1	MIRT507850
ASGR2	0.846153846	-16.5	MIRT504334
ASGR2	0.846153846	-16.5	MIRT504334
PTPRD	0.846153846	-17.6	MIRT555142
AHNAK2	0.846153846	-17.7	MIRT568574
SPRED1	0.846153846	-19	MIRT140152
TRIM35	0.846153846	-20.1	MIRT518091
UBR3	0.846153846	-17.9	MIRT086013
RAB3IP	0.846153846	-18.6	MIRT274745
RUNX1T1	0.846153846	-18.7	MIRT546623
RUNX1T1	0.846153846	-18.7	MIRT546623
CMTM4	0.846153846	-21.3	MIRT568021
CMTM4	0.846153846	-21.3	MIRT568021
NNT	0.846153846	-17.4	MIRT518994
PRICKLE2	0.846153846	-17.1	MIRT501502
NNT	0.846153846	-17.4	MIRT518994
AP2B1	0.846153846	-20.7	MIRT743291
AP2B1	0.846153846	-17	MIRT743291
AP2B1	0.846153846	-20.7	MIRT743291
AP2B1	0.846153846	-17	MIRT743291
AP2B1	0.846153846	-20.7	MIRT743291
AP2B1	0.846153846	-17	MIRT743291
ANKRD36	0.846153846	-16.3	MIRT646595
NNT	0.846153846	-17.4	MIRT518994

PHKA1	0.846153846	-15.8	MIRT506192
NNT	0.846153846	-17.4	MIRT518994
PRICKLE2	0.846153846	-17.1	MIRT501502
NFIA	0.846153846	-16	MIRT003928
AP2B1	0.846153846	-20.7	MIRT743291
AP2B1	0.846153846	-17	MIRT743291
WNK3	0.846153846	-19.4	MIRT564989
WNK3	0.846153846	-19.4	MIRT564989
HSPA4L	0.846153846	-15.1	MIRT211317
CARD10	0.846153846	-22	MIRT479989
ARHGAP32	0.846153846	-18.7	MIRT714438
TNRC6B	0.846153846	-16.7	MIRT158533
TNRC6B	0.846153846	-16.8	MIRT158533
CRIM1	0.846153846	-17.3	MIRT302619
ANKHD1	0.846153846	-22.5	MIRT042445
ANKHD1	0.846153846	-17.8	MIRT042445
CEP55	0.846153846	-18.6	MIRT057518
FGFR4	0.846153846	-19.2	MIRT460220
NOTCH2	0.846153846	-16.8	MIRT655812
ANKHD1	0.846153846	-17.8	MIRT042445
CLIP4	0.846153846	-18.5	MIRT548806
ZFHX4	0.846153846	-18	MIRT106300
ATAD5	0.846153846	-18.7	MIRT509690
L2HGDH	0.846153846	-18.1	MIRT500093
CSNK1E	0.846153846	-17	MIRT453750
GABPA	0.846153846	-16.6	MIRT297782
CDK17	0.846153846	-16.9	MIRT548949
PHKA1	0.846153846	-15.8	MIRT506192
SRPRA	0.846153846	-17.8	MIRT500935
KMT2D	0.846153846	-23.9	MIRT743303
FASN	0.846153846	-18.2	MIRT053106
RUNX1T1	0.846153846	-18.7	MIRT546623
KIF5B	0.846153846	-17.4	MIRT502148
CRKL	0.846153846	-18.9	MIRT367269
PDE4D	0.846153846	-17.8	MIRT471492
USP15	0.846153846	-17.3	MIRT066316
SYNRG	0.846153846	-19.7	MIRT725132
AHNAK2	0.846153846	-17.7	MIRT568574

KANK1	0.846153846	-17.9	MIRT556855
FBXL18	0.846153846	-18.6	MIRT743299
PRICKLE2	0.846153846	-17.1	MIRT501502
ARHGAP32	0.846153846	-18.7	MIRT714438
ADORA3	0.846153846	-16.6	MIRT717461
ALDH3B1	0.846153846	-18.4	MIRT451835
PLEKHA1	0.846153846	-18.1	MIRT055818
WNK3	0.846153846	-19.4	MIRT564989
ODF2L	0.846153846	-14.7	MIRT499452
GOSR1	0.846153846	-16.6	MIRT076796
GOSR1	0.846153846	-16.6	MIRT076796
TNRC6B	0.846153846	-16.8	MIRT158533
MINK1	0.846153846	-21.9	MIRT767957
MINK1	0.846153846	-21.3	MIRT767957
AP2B1	0.846153846	-20.7	MIRT743291
AP2B1	0.846153846	-17	MIRT743291
ALDH3B1	0.846153846	-18.4	MIRT451835
CHIC1	0.846153846	-17.3	MIRT327967
PTPRD	0.846153846	-17.6	MIRT555142
TRAK1	0.846153846	-18.1	MIRT614698
TPM3	0.846153846	-18.1	MIRT553439
TPM3	0.846153846	-18.1	MIRT553439
TPM3	0.846153846	-18.1	MIRT553439
TUBB2A	0.846153846	-15.8	MIRT561400
PTCH1	0.846153846	-18.9	MIRT438750
ZBTB34	0.846153846	-18.4	MIRT107222
MTFR1L	0.846153846	-20.1	MIRT066297
NFIA	0.846153846	-16	MIRT003928
TLK1	0.846153846	-17.4	MIRT570466
LANCL1	0.846153846	-17.3	MIRT562027
LANCL1	0.846153846	-17.3	MIRT562027
ARHGAP32	0.846153846	-18.7	MIRT714438
SLCO3A1	0.846153846	-17.2	MIRT282538
NFIA	0.846153846	-16	MIRT003928
NFIA	0.846153846	-16	MIRT003928
TNRC6B	0.846153846	-16.7	MIRT158533
TNRC6B	0.846153846	-16.8	MIRT158533
DCAF17	0.846153846	-21.1	MIRT680982

CDK17	0.846153846	-16.9	MIRT548949
PHKA1	0.846153846	-15.8	MIRT506192
SRPRA	0.846153846	-17.8	MIRT500935
ANKHD1	0.846153846	-17.8	MIRT042445
RUNX1T1	0.846153846	-18.7	MIRT546623
RUNX1T1	0.846153846	-18.7	MIRT546623
RUNX1T1	0.846153846	-18.7	MIRT546623
RUNX1T1	0.846153846	-18.7	MIRT546623
RUNX1T1	0.846153846	-18.7	MIRT546623
APP	0.846153846	-16.3	MIRT543079
SETD1B	0.871794872	-20	MIRT270556
SETD1B	0.871794872	-20	MIRT270556
KANK1	0.884615385	-19.9	MIRT556855
USP15	0.884615385	-17.7	MIRT066316
HCFC2	0.892307692	-18.1	MIRT547996
MINK1	0.897435897	-22.4	MIRT767957
MINK1	0.897435897	-22.4	MIRT767957
MINK1	0.897435897	-22.4	MIRT767957
PTPRD	0.897435897	-18	MIRT555142
PTPRD	0.897435897	-18	MIRT555142
PTPRD	0.897435897	-18	MIRT555142
PTPRD	0.897435897	-18	MIRT555142
PTPRD	0.897435897	-18	MIRT555142
TTLL5	0.914529915	-20.6	MIRT571283
KANK1	0.923076923	-19.9	MIRT556855
TRAK1	0.923076923	-18.1	MIRT614698
TPM3	0.923076923	-17.1	MIRT553439
TPM3	0.923076923	-17.9	MIRT553439
TPM3	0.923076923	-17.1	MIRT553439
TPM3	0.923076923	-17.9	MIRT553439
RAB3IP	0.923076923	-18.6	MIRT274745
ANKMY1	0.923076923	-19.2	MIRT618896
ANKMY1	0.923076923	-19.2	MIRT618896
TMEM161B	0.923076923	-19.7	MIRT553561
AMOTL1	0.923076923	-26	MIRT061250
RAB15	0.923076923	-16.8	MIRT716432
ANKMY1	0.923076923	-18.5	MIRT618896
PSAT1	0.923076923	-17.6	MIRT175237

PTPRD	0.923076923	-18	MIRT555142
PTPRD	0.923076923	-18	MIRT555142
PTPRD	0.923076923	-17.6	MIRT555142
GNAT1	0.923076923	-21.9	MIRT540843
DOCK11	0.923076923	-16.8	MIRT460434
PRSS21	0.923076923	-20.6	MIRT543525
CSNK1E	0.923076923	-17.9	MIRT453750
KANK1	0.923076923	-19.9	MIRT556855
TMEM161B	0.923076923	-19.7	MIRT553561
TPM3	0.923076923	-17.1	MIRT553439
MINK1	0.923076923	-23.2	MIRT767957
MINK1	0.923076923	-23.2	MIRT767957
RAB3IP	0.923076923	-18.6	MIRT274745
RAB3IP	0.923076923	-18.6	MIRT274745
OGT	0.923076923	-18.7	MIRT110057
RAB15	0.923076923	-16.8	MIRT716432
PRKAA1	0.923076923	-17.9	MIRT555225
RTN4	0.923076923	-17.6	MIRT546639
FGFR4	0.923076923	-19.2	MIRT460220
DOCK11	0.923076923	-16.8	MIRT460434
MINK1	0.923076923	-23.2	MIRT767957
PHC3	0.923076923	-18.5	MIRT542808
CMTM4	0.923076923	-21.3	MIRT568021
CLSPN	0.923076923	-20.5	MIRT544912
TRAK1	0.923076923	-18.1	MIRT614698
TRAK1	0.923076923	-18.1	MIRT614698
TRAK1	0.923076923	-18.1	MIRT614698
CCNE2	0.923076923	-20.1	MIRT507850
GOSR1	0.923076923	-15.7	MIRT076796
RAB15	0.923076923	-16.8	MIRT716432
TRAK1	0.923076923	-18.1	MIRT614698
TNRC6B	0.923076923	-19.1	MIRT158533
KANK1	0.923076923	-19.9	MIRT556855
PNISR	0.923076923	-16.6	MIRT042438
ANKMY1	0.923076923	-19.2	MIRT618896
ANKHD1	0.923076923	-20.3	MIRT042445
ANKMY1	0.923076923	-19.2	MIRT618896
ASH1L	0.923076923	-19.1	MIRT549276

ASH1L	0.923076923	-17.4	MIRT549276
MTFR1L	0.923076923	-20.1	MIRT066297
PSAT1	0.923076923	-17.6	MIRT175237
CLSPN	0.923076923	-20.5	MIRT544912
RAB3IP	0.923076923	-18.6	MIRT274745
RUSF1	0.923076923	-18.3	MIRT743294
ATG9A	0.923076923	-21.5	MIRT503143
PHC3	0.923076923	-18	MIRT542808
DCAF17	0.923076923	-21.1	MIRT680982
CSNK1E	0.923076923	-17.9	MIRT453750
FGFR4	0.923076923	-19.2	MIRT460220
CDC14A	0.923076923	-17.6	MIRT000654
MAP4K2	0.923076923	-18.3	MIRT545186
CCIN	0.923076923	-20.4	MIRT042434
PRKAA1	0.923076923	-17.9	MIRT555225
PNPLA6	0.923076923	-17.7	MIRT082989
SEC61A1	0.923076923	-19.2	MIRT743310
RTN4	0.923076923	-17.6	MIRT546639
RAB15	0.923076923	-16.8	MIRT716432
CLSPN	0.923076923	-20.5	MIRT544912
PDE4D	0.923076923	-17.8	MIRT471492
TRAK1	0.923076923	-18.1	MIRT614698
TMEM161B	0.923076923	-19.7	MIRT553561
KANK1	0.923076923	-19.9	MIRT556855
PRKAA1	0.923076923	-17.9	MIRT555225
PDE4D	0.923076923	-17.8	MIRT471492
TPM3	0.923076923	-17.1	MIRT553439
TPM3	0.923076923	-17.1	MIRT553439
RAB3IP	0.923076923	-18.6	MIRT274745
MINK1	0.923076923	-23.2	MIRT767957
ZNF284	0.923076923	-16.6	MIRT785523
PTPRD	0.923076923	-18	MIRT555142
TUBB2A	0.923076923	-18.7	MIRT561400
MTFR1L	0.923076923	-20.1	MIRT066297
PDE4D	0.923076923	-17.8	MIRT471492
APP	0.923076923	-16.3	MIRT543079
TNRC6B	0.923076923	-19.1	MIRT158533
PDE4D	0.923076923	-17.8	MIRT471492

PNPLA6	0.923076923	-17.7	MIRT082989
PDE4D	0.923076923	-17.8	MIRT471492
PDE4D	0.923076923	-17.8	MIRT471492
RUNX1T1	0.923076923	-18.7	MIRT546623
RUNX1T1	0.923076923	-18.7	MIRT546623
RUNX1T1	0.923076923	-18.7	MIRT546623
HSPE1-MOB4	0.923076923	-16.7	MIRT204597
RASEF	0.948717949	-21.8	MIRT566120
ANKRD17	0.961538462	-21.2	MIRT042449
AMOTL1	0.961538462	-26	MIRT061250
KIF5B	0.969230769	-18	MIRT502148
TRAP1	1	-21	MIRT042452
ANKMY1	1	-19.2	MIRT618896
PHLPP2	1	-19.2	MIRT547132
SLC35E2B	1	-24.4	MIRT713419
PHC3	1	-18	MIRT542808
ANKMY1	1	-19.2	MIRT618896
DIAPH1	1	-17.5	MIRT743298
ANKRD17	1	-21.2	MIRT042449
MINK1	1	-21.3	MIRT767957
MINK1	1	-21.3	MIRT767957
SESTD1	1	-23.8	MIRT505690
ANKRD17	1	-21.2	MIRT042449
CCDC80	1	-20.3	MIRT564552
CCDC80	1	-20.3	MIRT564552
GTF2A1	1	-24.5	MIRT042458
ASH1L	1	-19.1	MIRT549276
SACS	1	-21.5	MIRT042454
ASH1L	1	-19.1	MIRT549276
ASH1L	1	-19.1	MIRT549276
MINK1	1	-21.3	MIRT767957
ASH1L	1	-19.1	MIRT549276
TRAP1	1	-21	MIRT042452
KMT2D	1	-23.9	MIRT743303
ASH1L	1	-19.1	MIRT549276
ASH1L	1	-19.1	MIRT549276
SLC35E2B	1	-24.4	MIRT713419
EPM2AIP1	1	-22.1	MIRT551379

EPM2AIP1	1	-19.2	MIRT551379
PHLPP2	1	-19.2	MIRT547132
ANKRD17	1	-21.2	MIRT042449
GTF2A1	1	-24.5	MIRT042458
TRAP1	1	-21	MIRT042452
RIF1	1	-21.4	MIRT571428
DNAJC10	1	-17.8	MIRT552035
WNK3	1	-19.4	MIRT564989
IFIH1	1	-22.5	MIRT042455
IFIH1	1	-19.3	MIRT042455
ITGA2	1	-19.7	MIRT313679
GOSR1	1	-16.6	MIRT076796
DIAPH1	1	-17.5	MIRT743298
MYB	1	-19.4	MIRT005886
SNTB2	1	-23.6	MIRT075256
TLK1	1	-17.4	MIRT570466
ASH1L	1	-19.1	MIRT549276
CCNF	1	-19.4	MIRT000653
SIDT2	1	-20.1	MIRT443811
TPM3	1	-17.1	MIRT553439
ATG9A	1	-21.5	MIRT503143
MTFR1L	1	-20.1	MIRT066297
SLC35E2B	1	-24.4	MIRT713419
MYB	1	-19.4	MIRT005886
MYB	1	-19.4	MIRT005886
TLK1	1	-17.4	MIRT570466
MYB	1	-19.4	MIRT005886
MYB	1	-19.4	MIRT005886
MYB	1	-19.4	MIRT005886
MYB	1	-19.4	MIRT005886
MYB	1	-19.4	MIRT005886
RIF1	1	-21.4	MIRT571428
RIF1	1	-21.4	MIRT571428
RIF1	1	-21.4	MIRT571428
CLSPN	1	-20.5	MIRT544912
USP15	1	-17.3	MIRT066316

Supplementary Table6: Prediction results of target genes of hsa-miR-424-5p based on the miRPath dataset (only predictions with experimental support were retained).

Target	Evidence
ANLN	experimental (strong) + experimental (all) + predicted (union)
ATF6	experimental (strong) + experimental (all) + predicted (intersection) + predicted (union)
CCND1	experimental (strong) + experimental (all) + predicted (intersection) + predicted (union)
CCND3	experimental (strong) + experimental (all) + predicted (intersection) + predicted (union)
CCNE1	experimental (strong) + experimental (all) + predicted (intersection) + predicted (union)
CCNF	experimental (strong) + experimental (all) + predicted (intersection) + predicted (union)
CDC14A	experimental (strong) + experimental (all) + predicted (intersection) + predicted (union)
CDC25A	experimental (strong) + experimental (all) + predicted (intersection) + predicted (union)
CDK6	experimental (strong) + experimental (all) + predicted (intersection) + predicted (union)
CDX2	experimental (strong) + experimental (all) + predicted (intersection) + predicted (union)
CHEK1	experimental (strong) + experimental (all) + predicted (intersection) + predicted (union)
CUL2	experimental (strong) + experimental (all)
FASN	experimental (strong) + experimental (all) + predicted (intersection) + predicted (union)
FGF2	experimental (strong) + experimental (all) + predicted (intersection) + predicted (union)
FGFR1	experimental (strong) + experimental (all) + predicted (intersection) + predicted (union)
HIF1A	experimental (strong) + experimental (all) + predicted (union)
KDM5B	experimental (strong) + experimental (all) + predicted (intersection) + predicted (union)
KIF23	experimental (strong) + experimental (all) + predicted (intersection) + predicted (union)
MAP2K1	experimental (strong) + experimental (all) + predicted (intersection) + predicted (union)
MYB	experimental (strong) + experimental (all) + predicted (intersection) + predicted (union)
NFIA	experimental (strong) + experimental (all) + predicted (union)
PLAG1	experimental (strong) + experimental (all) + predicted (intersection) + predicted (union)
PTCH1	experimental (strong) + experimental (all) + predicted (intersection) + predicted (union)
SIAH1	experimental (strong) + experimental (all)
SMAD3	experimental (strong) + experimental (all) + predicted (intersection) + predicted (union)
SMAD7	experimental (strong) + experimental (all) + predicted (intersection) + predicted (union)
SOCS2	experimental (strong) + experimental (all)
SOCS6	experimental (strong) + experimental (all) + predicted (intersection) + predicted (union)
SPI1	experimental (strong) + experimental (all)
TGFBR3	experimental (strong) + experimental (all) + predicted (intersection) + predicted (union)
WEE1	experimental (strong) + experimental (all) + predicted (intersection) + predicted (union)

Supplementary Table7: Prediction results of lncRNA targeting hsa-miR-424-5p based on the starBase dataset.

	miRNAname	geneName	geneType
1	hsa-miR-424-5p	AL391244.1	processed_transcript
4	hsa-miR-424-5p	AL031282.2	processed_transcript

5	hsa-miR-424-5p	AL031281.2	lincRNA
6	hsa-miR-424-5p	SNHG12	antisense
7	hsa-miR-424-5p	RP6-206I17.2	lincRNA
9	hsa-miR-424-5p	MIR29B2CHG	lincRNA
10	hsa-miR-424-5p	AL359924.1	antisense
11	hsa-miR-424-5p	FGD5-AS1	antisense
12	hsa-miR-424-5p	LINC02035	lincRNA
13	hsa-miR-424-5p	AC098864.1	antisense
14	hsa-miR-424-5p	AC114980.1	sense_intronic
15	hsa-miR-424-5p	LINC01184	lincRNA
16	hsa-miR-424-5p	AC021078.1	processed_transcript
18	hsa-miR-424-5p	HCG18	antisense
21	hsa-miR-424-5p	AC003092.1	lincRNA
22	hsa-miR-424-5p	AC079781.5	processed_transcript
26	hsa-miR-424-5p	STAG3L5P-PVRIG2P-PILRB	processed_transcript
27	hsa-miR-424-5p	NUTM2B-AS1	antisense
28	hsa-miR-424-5p	NUTM2A-AS1	antisense
29	hsa-miR-424-5p	SNHG1	processed_transcript
32	hsa-miR-424-5p	NEAT1	lincRNA
34	hsa-miR-424-5p	AC020978.7	sense_overlapping
35	hsa-miR-424-5p	AC009022.1	processed_transcript
36	hsa-miR-424-5p	MIR497HG	antisense
37	hsa-miR-424-5p	AC016876.1	antisense
39	hsa-miR-424-5p	AC018628.1	TEC
40	hsa-miR-424-5p	SNHG25	lincRNA
41	hsa-miR-424-5p	SNHG16	processed_transcript
44	hsa-miR-424-5p	LINC00662	lincRNA
45	hsa-miR-424-5p	AC008555.8	TEC
47	hsa-miR-424-5p	AC021092.1	antisense
48	hsa-miR-424-5p	AC005261.1	lincRNA
49	hsa-miR-424-5p	MZF1-AS1	antisense
50	hsa-miR-424-5p	LINC00649	antisense
51	hsa-miR-424-5p	AP000317.2	processed_transcript
53	hsa-miR-424-5p	MCM3AP-AS1	antisense
55	hsa-miR-424-5p	AC002470.2	processed_transcript
57	hsa-miR-424-5p	AC004656.1	sense_overlapping
62	hsa-miR-424-5p	XIST	lincRNA
64	hsa-miR-424-5p	AL035425.3	sense_overlapping

65	hsa-miR-424-5p	MIR503HG	lincRNA

supplementary Table8: Prediction results for drugs targeting PLAUR based on the DrugMatrix dataset.

Term	Overlap	P-value	Adjusted P-value	Combined Score	Genes
Luronit CTD 00006106	1/37	0.00185	0.033366	125618.9	PLAUR
AMILORIDE CTD 00005369	1/48	0.0024	0.033366	120356.5	PLAUR
choline CTD 00005662	1/52	0.0026	0.033366	118735.7	PLAUR
MK 886 CTD 00002517	1/52	0.0026	0.033366	118735.7	PLAUR
valsartan CTD 00002971	1/64	0.0032	0.033366	114524.7	PLAUR
chlorprothixene PC3 UP	1/71	0.00355	0.033366	112416	PLAUR
biochanin A CTD 00000149	1/84	0.0042	0.033366	108994	PLAUR
Bexarotene CTD 00003225	1/92	0.0046	0.033366	107139.1	PLAUR
ciclopirox MCF7 UP	1/103	0.00515	0.033366	104832.8	PLAUR
phenytoin CTD 00006527	1/110	0.0055	0.033366	103488.1	PLAUR
LITHOCHOLIC ACID CTD 00006221	1/116	0.0058	0.033366	102400.8	PLAUR
1,4-chrysenequinone PC3 UP	1/119	0.00595	0.033366	101877.7	PLAUR
colchicine HL60 UP	1/132	0.0066	0.033366	99751.22	PLAUR
3,4-DICHLOROANILINE CTD 00000754	1/136	0.0068	0.033366	99138.14	PLAUR
pyrvinium HL60 UP	1/137	0.00685	0.033366	98987.63	PLAUR
thalidomide CTD 00006858	1/142	0.0071	0.033366	98250.87	PLAUR
diuron CTD 00005864	1/144	0.0072	0.033366	97963.27	PLAUR
etoposide HL60 UP	1/147	0.00735	0.033366	97539.11	PLAUR
Vanadic sulfate CTD 00001628	1/158	0.0079	0.033366	96053.21	PLAUR
luteolin HL60 UP	1/159	0.00795	0.033366	95923.19	PLAUR
ellipticine HL60 DOWN	1/161	0.00805	0.033366	95665.53	PLAUR
5109870 MCF7 UP	1/171	0.00855	0.033366	94422.42	PLAUR
niclosamide HL60 UP	1/178	0.0089	0.033366	93593.83	PLAUR
1,9-Pyrazoloanthrone CTD 00003948	1/182	0.0091	0.033366	93134.52	PLAUR
folic acid CTD 00005997	1/195	0.00975	0.034059	91707.02	PLAUR
Capsaicin CTD 00005570	1/207	0.01035	0.034059	90469.42	PLAUR
Sodium dichromate CTD 00000827	1/209	0.01045	0.034059	90269.98	PLAUR
PHENCYCLIDINE CTD 00005881	1/227	0.01135	0.035048	88554.31	PLAUR
nitrofural PC3 DOWN	1/231	0.01155	0.035048	88191.08	PLAUR
PD 98059 CTD 00003206	1/263	0.01315	0.037631	85487.71	PLAUR

OCHRATOXIN A CTD 00001202	1/298	0.0149	0.037631	82874.54	PLAUR
podophyllotoxin HL60 UP	1/306	0.0153	0.037631	82319.16	PLAUR
Fulvestrant CTD 00002740	1/311	0.01555	0.037631	81979.15	PLAUR
pergolide HL60 UP	1/312	0.0156	0.037631	81911.78	PLAUR
N-Acetyl-L-cysteine CTD 00005305	1/316	0.0158	0.037631	81644.38	PLAUR
MG-262 PC3 UP	1/320	0.016	0.037631	81380.24	PLAUR
8-azaguanine PC3 UP	1/323	0.01615	0.037631	81184.22	PLAUR
cycloheximide CTD 00005731	1/325	0.01625	0.037631	81054.52	PLAUR
mebendazole HL60 UP	1/346	0.0173	0.039036	79737.39	PLAUR
vorinostat PC3 DOWN	1/355	0.01775	0.03905	79196.41	PLAUR
nocodazole HL60 UP	1/373	0.01865	0.039704	78153.08	PLAUR
ouabain HL60 UP	1/379	0.01895	0.039704	77816.08	PLAUR
piperlongumine HL60 DOWN	1/389	0.01945	0.039804	77265.69	PLAUR
alprostadil HL60 UP	1/409	0.02045	0.040068	76204.67	PLAUR
digitoxigenin HL60 UP	1/421	0.02105	0.040068	75591.81	PLAUR
dexamethasone CTD 00005779	1/422	0.0211	0.040068	75541.5	PLAUR
8-azaguanine HL60 UP	1/428	0.0214	0.040068	75242.04	PLAUR
digoxin MCF7 DOWN	1/449	0.02245	0.041158	74224.82	PLAUR
3-(1-methylpyrrolidin-2-yl)pyridine CTD 00006393	1/500	0.025	0.04454	71933.28	PLAUR
oxygen CTD 00006454	1/525	0.02625	0.04454	70890.86	PLAUR
curcumin CTD 00000663	1/529	0.02645	0.04454	70728.51	PLAUR
trichostatin A HL60 DOWN	1/543	0.02715	0.04454	70169.42	PLAUR
piroxicam CTD 00006571	1/550	0.0275	0.04454	69895.04	PLAUR
POTASSIUM DICHROMATE CTD 00006598	1/557	0.02785	0.04454	69623.99	PLAUR
aspirin CTD 00005447	1/562	0.0281	0.04454	69432.38	PLAUR
vorinostat HL60 DOWN	1/569	0.02845	0.04454	69166.84	PLAUR
NICKEL SULFATE CTD 00001417	1/577	0.02885	0.04454	68867.18	PLAUR
azacitidine PC3 DOWN	1/595	0.02975	0.045138	68207.26	PLAUR
benzene CTD 00005481	1/616	0.0308	0.045613	67461.1	PLAUR
ambroxol PC3 DOWN	1/622	0.0311	0.045613	67252.38	PLAUR
raloxifene CTD 00007367	1/687	0.03435	0.049109	65107.19	PLAUR
scriptaid PC3 DOWN	1/692	0.0346	0.049109	64950.32	PLAUR
metronidazole PC3 DOWN	1/745	0.03725	0.052031	63351.04	PLAUR
cephaeline HL60 UP	1/767	0.03835	0.052731	62718.92	PLAUR
ampyrone HL60 DOWN	1/781	0.03905	0.052867	62325.63	PLAUR
emetine HL60 UP	1/849	0.04245	0.056052	60506.3	PLAUR
lycorine HL60 UP	1/862	0.0431	0.056052	60174.41	PLAUR
Cianidanol CTD 00005600	1/871	0.04355	0.056052	59947.42	PLAUR

Bortezomib CTD 00003736	1/879	0.04395	0.056052	59747.53	PLAUR
cimetidine PC3 DOWN	1/953	0.04765	0.059903	57976.73	PLAUR
trichostatin A PC3 DOWN	1/1133	0.05665	0.069789	54164.65	PLAUR
anisomycin HL60 UP	1/1142	0.0571	0.069789	53989.61	PLAUR
testosterone CTD 00006844	1/1248	0.0624	0.075222	52021.68	PLAUR
Arsenenous acid CTD 00000922	1/1284	0.0642	0.076346	51389.56	PLAUR
TERT-BUTYL HYDROPEROXIDE CTD 00007349	1/1342	0.0671	0.07873	50405.98	PLAUR
alsterpaullone MCF7 DOWN	1/1469	0.07345	0.085047	48387.29	PLAUR
resveratrol CTD 00002483	1/1602	0.0801	0.091543	46445.43	PLAUR
ZINC CTD 00007011	1/1643	0.08215	0.092682	45878.02	PLAUR
progesterone CTD 00006624	1/1916	0.0958	0.106714	42415.94	PLAUR
calcitriol CTD 00005558	1/1959	0.09795	0.107745	41914.67	PLAUR
hydrogen peroxide CTD 00006118	1/2674	0.1337	0.145254	34862.66	PLAUR
7646-79-9 CTD 00000928	1/3095	0.15475	0.166073	31543.82	PLAUR
0175029-0000 PC3 DOWN	1/3326	0.1663	0.176318	29912.55	PLAUR
Tetradioxin CTD 00006848	1/3769	0.18845	0.197424	27088.3	PLAUR
Retinoic acid CTD 00006918	1/4259	0.21295	0.220466	24346.59	PLAUR
estradiol CTD 00005920	1/4337	0.21685	0.221893	23941.69	PLAUR
cyclosporin A CTD 00007121	1/4826	0.2413	0.244073	21573.11	PLAUR
Copper sulfate CTD 00007279	1/6017	0.30085	0.30085	16795.6	PLAUR
